# Selective transcriptional regulation by Myc: Experimental design and computational analysis of high-throughput sequencing data

**DOI:** 10.1016/j.dib.2015.02.003

**Published:** 2015-02-12

**Authors:** Mattia Pelizzola, Marco J. Morelli, Arianna Sabò, Theresia R. Kress, Stefano de Pretis, Bruno Amati

**Affiliations:** a Center for Genomic Science of IIT@SEMM, Fondazione Istituto Italiano di Tecnologia (IIT), Via Adamello 16, 20139 Milan, Italy; b Department of Experimental Oncology, European Institute of Oncology (IEO), Via Adamello 16, 20139 Milan, Italy

**Keywords:** B-cell lymphoma, Transcription, Post-translational histone modifications

## Abstract

The gene expression programs regulated by the Myc transcription factor were evaluated by integrated genome-wide profiling of Myc binding sites, chromatin marks and RNA expression in several biological models. Our results indicate that Myc directly drives selective transcriptional regulation, which in certain physiological conditions may indirectly lead to RNA amplification. Here, we illustrate in detail the experimental design concerning the high-throughput sequencing data associated with our study (Sabò et al., Nature. (2014) 511:488–492) and the R scripts used for their computational analysis.

**Specifications Table**

**Table:** 

Organism/cell line/tissue	*Human (P493-6 B cells), mouse (Eμ-myc B cells, 3T9 fibroblasts)*

Sex	*Not applicable*
Sequencer or array type	*Illumina Hi-Seq 2000*
Data format	*Raw and analyzed*
Experimental factors	***Eµ-*****myc**:* wild type B-cells (Control, “C”), Eµ-*myc* transgenic B-cells not yet transformed (Pre-tumoral, “P”), and lymphoma cells (Tumor, “T”)*
	***3T9.Serum**: 3T9 fibroblasts serum starved (t0 h) or released for 1 (t1 h) or 2 (t2 h) hours*
	***3T9.mycER**: MycER-infected 3T9 fibroblasts untreated (0 hOHT) or treated for different periods of times with OHT (4, 8, 16 hOHT) to activate the MycER chimera.*
	***P493**: P493-6 cells treated with Tetracycline (Myc transgene repressed) for 72 h (t0) and then released in fresh medium without Tetracycline (allowing expression of the transgene) for 1 h (t1 h), 24 h (t24 h) or several passages (“High Myc”). P493-6 cells treated with Tetracycline (Myc transgene repressed) plus OHT (endogenous Myc activated: “Low Myc”)*
Experimental features	*Cells with the indicated genotype/treatment were used for ChIP-Seq (for Myc, RNAPII, H3K4me3, H3K4me1, H3K27ac), totRNA-Seq, 4sU-RNA-Seq or DNAse-Seq experiments, as reported*
Consent	*n/a*
Sample source location	*Milan, Italy*

## 1 Direct link to deposited data

http://www.ncbi.nlm.nih.gov/geo/query/acc.cgi?acc=GSE51011.

## 2 Experimental design

The Gene Expression Omnibus (GEO) Series GSE51011 contains 94 high-throughput sequencing samples associated with the Sabò et al. study [1]. These samples cover different –omics datasets (ChIP-Seq for transcription factors and post-translational histone modifications, RNA-Seq, 4sU-Seq and DNAseI-Seq) produced in different organisms and biological systems. Some of the samples have to be used as references for other samples: for example, as inputs in the ChIP-seq peak calling procedure, or as baselines for the identification of differentially expressed genes. To help navigating through these data we collected the most relevant associated metadata in Table 1.

The different biological systems used in the study allowed the analysis of the effects of modulation of Myc levels in vitro and in vivo, both at physiological and pathological levels. In the Eμ-*myc* model [2], Myc overexpression was achieved in vivo specifically in the mouse B-cell compartment, where it causes lymphoma development. This model system gave access to primary wild type B-cells (Control, “C”), Eµ-*myc* transgenic B-cells not yet transformed (Pre-tumoral, “P”), and lymphoma tumoral cells (Tumor, “T”). Modulation of Myc expression in human B-cells was obtained in a time-controlled manner in vitro in the cell line P493-6 [3], harboring a tet-regulated Myc transgene. A line of mouse 3T9 fibroblasts was also used in which endogenous c-*myc* was modulated from low basal levels (in conditions of serum starvation) to mitogen-induced levels (upon serum stimulation). In the same cells, we expressed a conditionally active MycER chimaera, allowing us to induce active Myc at supra-physiological levels through administration of OHT to the culture medium.

## 3 Data analysis: Source code design and installation

In addition to the methods in the original publication [1], the source code used for the computational analysis of the high-throughput sequencing data is available as supplemental material of this manuscript.

### 3.1 R/Bioconductor and the compEpiTools package

The source code is entirely written using R, an open-source language and environment for statistical computing and graphics. In particular, several of the scripts developed for this study take advantage of R packages developed within the Bioconductor project [4], which currently counts more than 700 packages contributed from the scientific community, mostly dedicated to the analysis of high-throughput biological data. In the Bioconductor spirit, most of the scripts developed for this study were included in an R package (compEpiTools), which was recently approved as part of that project and is available on the Bioconductor website at the following URL: http://www.bioconductor.org/packages/release/bioc/html/compEpiTools.html. To ensure complete reproducibility, we include here the original version of the compEpiTools package (v0.1) preceding the submission to Bioconductor, which was the one actually used for the computational analysis of the published data. Importantly, compEpiTools (both v0.1 and following versions) is totally compliant with the Bioconductor computational infrastructures, and therefore the results generated here are highly compatible with the other tools offered by Bioconductor.

From here on, R commands will be indicated enclosed within quotes (e.g. ‘load’), while file and folder names will be indicated in italic (e.g. *file*1*.txt*).

### 3.2 Description of source code files

The source code (*saboEtAl*2014*_sourceCode.zip*) is composed by 6 files and two folders:

• *compEpiTools_*0.1*.tar.gz*
  An R package (requires R_3.0.2) containing most of the functions and methods used for the data analysis, including documentation and examples. The package is a preliminary version of a package currently available and maintained on the Bioconductor project (http://www.bioconductor.org/packages/release/bioc/html/compEpiTools.html). While the package version available on the Bioconductor web site is continuously updated, we strongly recommend using the attached version 0.1 to exactly reproduce the analyses documented here. The compEpiTools version available on Bioconductor can be used as reference for running examples of individual methods that might be missing in the 0.1 compEpiTools version. The compEpiTools version 0.1 package can be installed on Linux and MacOS systems, using the ‘install.packages’ R command. Please refer to the R (http://www.r-project.org/) and Bioconductor (http://www.bioconductor.org/) web sites for documentation and tutorials on the R language and common Bioconductor infrastructure (e.g. the GRanges object) and methods.
• *filemapping_GEO.R*
  Contains virtual links to the sequencing data indicated in Table 1 and processing steps used to transform peak lists in GRanges R objects, which were saved in the R binary file *peaksRef.rda* in the *data* folder.
• *analysisEnvironment.R*
  An R script, including a number of additional compEpiTools functions and methods not contained in *compEpiTools_*0.1*.tar.gz*. This script can be called with the ‘source’ R command when initializing the R session.
• *saboEtAl*2014*_Figures.R*
  An R script containing the code used to generate the main figures resulting from computational analyses in the published paper [1].
• *saboEtAl*2014*_ExtData.R*
  An R script containing the code used to generate the extended figures resulting from computational analyses in the published paper [1].
• *saboEtAl*2014*_ExtData*10*.R*
  An R script containing the code used to generate extended figure 10 in the published paper [1].
• *data* folder
  A folder containing the input and output data, formatted as R objects or text files.
• *figures* folder
  A folder containing the figures resulting from the computational data analysis, which were used as panels to assemble the main and extended figures in the published paper [1].

This file is also available at the following URL: http://genomics.iit.it/supplementalData/SaboNature2014. In case updated versions will be necessary they will be released there, while the original zip file will always be available.

### 3.3 Getting started

The file *filemapping_GEO.R* allows matching of the GEO samples listed in Table 1 with the corresponding computational objects. In particular, the code shows how genomic regions such as ChIP-Seq peaks and DNAseI-Seq hypersensitive sites were stored as GRange objects. A GRange is a basic Bioconductor infrastructure that minimally contains the chromosome assignments as well as the start and end nucleotide positions for a set of genomic regions. *filemapping_GEO.R* reports how the ChIP-seq peaks were processed, i.e. considering the filters on the associated p-values and the pooling of replicated experiments (see [1] methods section for a description of the actual peak calling procedure). The final lists of peaks were saved in the *peaksRef.rda* file available in the *data* folder. This is a binary file containing an R object (list of GRanges) and can be loaded into R using the ‘load’ R command.

The *analysisEnvironment.R* file contains a set of R commands needed for the setup of the working environment before starting to reproduce the analyses contained in the *saboEtAl*2014_* files. The ‘source’ R command can be used to execute the R commands included in *analysisEnvironment.R*. This uploads a number of data and additional functions into the R memory and executes processing steps described at the bottom of the *analysisEnvironment.R* file.

The *saboEtAl*2014*_Figures.R* file contains the R commands used to generate individual figures (or panels) as indicated throughout the code. The code can be copied and pasted in the R GUI (or in the command line shell) to obtain the resulting data or figure. Please note that some steps might depend on the execution of previous steps reported in the same file. The results were already incorporated in the *saboEtAl*2014*_Figures.R* file itself (in case of numbers) or included in the *figures* folder (in case of figures or figure panels).

The same logic applies to *saboEtAl*2014*_ExtData.R* and *saboEtAl*2014*_ExtData*10*.R* for the results reported in the extended figures.

The original FASTQ sequencing data were submitted to GEO (GSE51011 series) and are available there as SRA files. SRA files can be transformed back to FASTQ files using the fastq-dump tool (http://www.ncbi.nlm.nih.gov/Traces/sra/sra.cgi?view=toolkit_doc&f=fastq-dump). Finally, BAM files can be obtained by aligning the FASTQ files the reference genome indicated in Table 1, using BWA and TopHat aligners using default parameters for ChIP-seq and RNA-seq data, respectively.

## 4 RNAPII stalling index analysis

The RNAPII stalling index (SI) was determined relating the RNAPII ChIP-seq reads density in the region around the transcription start site (tss) to the density in the genebody (GB). Specifically, the SI was obtained as a ratio of the number of reads counted on RNAPII alignment files in the interval [tss−300 bp, tss+300] (TSS) and [tss+300, transcription end site+3000] (GB): SI=TSS/GB [5]. In literature, this quantity was referred alternatively as travelling ratio (TR=GB/TSS) [6]; yet, some studies refer as travelling ratio the ratio TSS/GB [7,8]. The stalling index was computed considering all transcripts whose length was above 600 bp having an RNAPII ChIP-seq peak on their TSS. Since the stalling index reflects the balance between two different effects (the amount of RNAPII loaded on the TSS of a gene, and the amount of RNAPII travelling on the genebody), the code in the R source file *saboEtAl*2014*_ExtData*10*.R* clarifies this point by separately plotting the TSS and GB read distributions along with the SI.

## Figures and Tables

**Table 1:** Key features of the 94 samples available in the GSE51011 series (rep=replicate, org=organism, mmu=mm9, hsa=hg18).

**Sample id**	**Sample name**	**Rep**	**Data type**	**Target**	**Org**	**Biological model**	**Input/baseline**
GSM1234471	Eu-myc.Myc.C.1	1/1	ChIP-Seq	Myc	mmu	Eμ-*myc*	GSM1395178
GSM1395178	Eu-myc.input.C1	1/1	ChIP-Seq	Myc	mmu	Eμ-*myc*	–
GSM1234472	Eu-myc.Myc.P.1	1/3	ChIP-Seq	Myc	mmu	Eμ-*myc*	GSM1395179
GSM1395179	Eu-myc.input.P1	1/1	ChIP-Seq	Myc	mmu	Eμ-*myc*	–
GSM1234473	Eu-myc.Myc.P.2	2/3	ChIP-Seq	Myc	mmu	Eμ-*myc*	GSM1234498
GSM1234474	Eu-myc.Myc.P.3	3/3	ChIP-Seq	Myc	mmu	Eμ-*myc*	GSM1234498
GSM1234475	Eu-myc.Myc.T.1	1/1	ChIP-Seq	Myc	mmu	Eμ-*myc*	GSM1234498
GSM1234476	Eu-myc.Myc.T.2	1/1	ChIP-Seq	Myc	mmu	Eμ-*myc*	GSM1234498
GSM1234477	Eu-myc.Myc.T.3	1/1	ChIP-Seq	Myc	mmu	Eμ-*myc*	GSM1234498
GSM1234478	Eu-myc.Pol2.C.1	1/1	ChIP-Seq	Pol2	mmu	Eμ-*myc*	GSM1234498
GSM1234479	Eu-myc.Pol2.P.1	1/1	ChIP-Seq	Pol2	mmu	Eμ-*myc*	GSM1234498
GSM1234480	Eu-myc.Pol2.T.1	1/1	ChIP-Seq	Pol2	mmu	Eμ-*myc*	GSM1234498
GSM1234481	Eu-myc.Pol2.T.2	1/1	ChIP-Seq	Pol2	mmu	Eμ-*myc*	GSM1234498
GSM1234482	Eu-myc.Pol2.T.3	1/1	ChIP-Seq	Pol2	mmu	Eμ-*myc*	GSM1234498
GSM1234483	Eu-myc.H3K4me3.C1	1/2	ChIP-Seq	H3K4me3	mmu	Eμ-*myc*	GSM1234498
GSM1234517	Eu-myc.H3K4me3.C2	2/2	ChIP-Seq	H3K4me3	mmu	Eμ-*myc*	GSM1234498
GSM1234484	Eu-myc.H3K4me3.P1	1/2	ChIP-Seq	H3K4me3	mmu	Eμ-*myc*	GSM1234498
GSM1234518	Eu-myc.H3K4me3.P2	2/2	ChIP-Seq	H3K4me3	mmu	Eμ-*myc*	GSM1234498
GSM1234485	Eu-myc.H3K4me3.T1	1/1	ChIP-Seq	H3K4me3	mmu	Eμ-*myc*	GSM1234498
GSM1234486	Eu-myc.H3K4me3.T2	1/1	ChIP-Seq	H3K4me3	mmu	Eμ-*myc*	GSM1234498
GSM1234487	Eu-myc.H3K4me3.T3	1/1	ChIP-Seq	H3K4me3	mmu	Eμ-*myc*	GSM1234498
GSM1234488	Eu-myc.H3K4me1.C1	1/2	ChIP-Seq	H3K4me1	mmu	Eμ-*myc*	GSM1234498
GSM1234519	Eu-myc.H3K4me1.C2	2/2	ChIP-Seq	H3K4me1	mmu	Eμ-*myc*	GSM1234498
GSM1234489	Eu-myc.H3K4me1.P1	1/2	ChIP-Seq	H3K4me1	mmu	Eμ-*myc*	GSM1234498
GSM1234520	Eu-myc.H3K4me1.P2	2/2	ChIP-Seq	H3K4me1	mmu	Eμ-*myc*	GSM1234498
GSM1234490	Eu-myc.H3K4me1.T1	1/1	ChIP-Seq	H3K4me1	mmu	Eμ-*myc*	GSM1234498
GSM1234491	Eu-myc.H3K4me1.T2	1/1	ChIP-Seq	H3K4me1	mmu	Eμ-*myc*	GSM1234498
GSM1234492	Eu-myc.H3K4me1.T3	1/1	ChIP-Seq	H3K4me1	mmu	Eμ-*myc*	GSM1234498
GSM1234493	Eu-myc.H3K27ac.C	1/1	ChIP-Seq	H3K27ac	mmu	Eμ-*myc*	GSM1234498
GSM1234494	Eu-myc.H3K27ac.P	1/1	ChIP-Seq	H3K27ac	mmu	Eμ-*myc*	GSM1234498
GSM1234495	Eu-myc.H3K27ac.T1	1/1	ChIP-Seq	H3K27ac	mmu	Eμ-*myc*	GSM1234498
GSM1234496	Eu-myc.H3K27ac.T2	1/1	ChIP-Seq	H3K27ac	mmu	Eμ-*myc*	GSM1234498
GSM1234497	Eu-myc.H3K27ac.T3	1/1	ChIP-Seq	H3K27ac	mmu	Eμ-*myc*	GSM1234498
GSM1234498	Eu-myc.input.CPT	1/1	ChIP-Seq	–	mmu	Eμ-*myc*	–
GSM1386348	3T9.Serum.Myc.t0h	1/1	ChIP-Seq	Myc	mmu	3T9.Serum	GSM1386351
GSM1386349	3T9.Serum.Myc.t1h	1/1	ChIP-Seq	Myc	mmu	3T9.Serum	GSM1386351
GSM1386350	3T9.Serum.Myc.t2h	1/1	ChIP-Seq	Myc	mmu	3T9.Serum	GSM1386351
GSM1386351	3T9.Serum.input	1/1	ChIP-Seq	–	mmu	3T9.Serum	–
GSM1234500	P493.Myc.LowMyc	1/1	ChIP-Seq	Myc	hsa	P493	GSM1386347
GSM1234501	P493.Myc.HighMyc	1/1	ChIP-Seq	Myc	hsa	P493	GSM1386347
GSM1234499	P493.Myc.t0h	1/1	ChIP-Seq	Myc	hsa	P493	GSM1386347
GSM1386342	P493.Myc.t1h	1/1	ChIP-Seq	Myc	hsa	P493	GSM1386347
GSM1386343	P493.Myc.t24h	1/1	ChIP-Seq	Myc	hsa	P493	GSM1386347
GSM1234502	P493.Pol2.t0h	1/1	ChIP-Seq	Pol2	hsa	P493	GSM1386347
GSM1386344	P493.Pol2.t24h	1/1	ChIP-Seq	Pol2	hsa	P493	GSM1386347
GSM1386345	P493.H3K4me3.t24h	1/1	ChIP-Seq	H3K4me3	hsa	P493	GSM1386347
GSM1386346	P493.H3K27ac.t24h	1/1	ChIP-Seq	H3K27ac	hsa	P493	GSM1386347
GSM1386347	P493.input	1/1	ChIP-Seq	–	hsa	P493	–
GSM1234505	3T9.mycER.Pol2.0hOHT	1/1	ChIP-Seq	Pol2	mmu	3T9.mycER	GSM1234507
GSM1234506	3T9.mycER.Pol2.4hOHT	1/1	ChIP-Seq	Pol2	mmu	3T9.mycER	GSM1234507
GSM1234507	3T9.mycER.input.Pol2	1/1	ChIP-Seq	-	mmu	3T9.mycER	-
GSM1234508	3T9.mycER.Myc.0hOHT	1/1	ChIP-Seq	Myc	mmu	3T9.mycER	GSM1234516
GSM1234509	3T9.mycER.Myc.4hOHT	1/1	ChIP-Seq	Myc	mmu	3T9.mycER	GSM1234516
GSM1234510	3T9.mycER.H3K4me3.0hOHT	1/1	ChIP-Seq	H3K4me3	mmu	3T9.mycER	GSM1234516
GSM1234511	3T9.mycER.H3K4me3.4hOHT	1/1	ChIP-Seq	H3K4me3	mmu	3T9.mycER	GSM1234516
GSM1234512	3T9.mycER.H3K4me1.0hOHT	1/1	ChIP-Seq	H3K4me1	mmu	3T9.mycER	GSM1234516
GSM1234513	3T9.mycER.H3K4me1.4hOHT	1/1	ChIP-Seq	H3K4me1	mmu	3T9.mycER	GSM1234516
GSM1234514	3T9.mycER.H3K27ac.0hOHT	1/1	ChIP-Seq	H3K27ac	mmu	3T9.mycER	GSM1234516
GSM1234515	3T9.mycER.H3K27ac.4hOHT	1/1	ChIP-Seq	H3K27ac	mmu	3T9.mycER	GSM1234516
GSM1234516	3T9.mycER.input.OHT	1/1	ChIP-Seq	–	mmu	3T9.mycER	–
GSM1234734	Eu-myc.RNAseq.C_1	1/4	RNA-Seq	totRNA	mmu	Eμ-*myc*	–
GSM1234735	Eu-myc.RNAseq.C_3	2/4	RNA-Seq	totRNA	mmu	Eμ-*myc*	–
GSM1234736	Eu-myc.RNAseq.C_4	3/4	RNA-Seq	totRNA	mmu	Eμ-*myc*	–
GSM1234737	Eu-myc.RNAseq.C_6	4/4	RNA-Seq	totRNA	mmu	Eμ-*myc*	–
GSM1234738	Eu-myc.RNAseq.P_2	1/4	RNA-Seq	totRNA	mmu	Eμ-*myc*	GSM1234734-7
GSM1234739	Eu-myc.RNAseq.P_3	2/4	RNA-Seq	totRNA	mmu	Eμ-*myc*	GSM1234734-7
GSM1234740	Eu-myc.RNAseq.P_4	3/4	RNA-Seq	totRNA	mmu	Eμ-*myc*	GSM1234734-7
GSM1234741	Eu-myc.RNAseq.P_5	4/4	RNA-Seq	totRNA	mmu	Eμ-*myc*	GSM1234734-7
GSM1234742	Eu-myc.RNAseq.T_1	1/1	RNA-Seq	totRNA	mmu	Eμ-*myc*	GSM1234734-7
GSM1234743	Eu-myc.RNAseq.T_2	1/1	RNA-Seq	totRNA	mmu	Eμ-*myc*	GSM1234734-7
GSM1234744	Eu-myc.RNAseq.T_3	1/1	RNA-Seq	totRNA	mmu	Eμ-*myc*	GSM1234734-7
GSM1234745	3T9.mycER.RNAseq.0hOHT_1	1/4	RNA-Seq	totRNA	mmu	3T9.mycER	–
GSM1234746	3T9.mycER.RNAseq.0hOHT_2	2/4	RNA-Seq	totRNA	mmu	3T9.mycER	–
GSM1234747	3T9.mycER.RNAseq.0hOHT_3	3/4	RNA-Seq	totRNA	mmu	3T9.mycER	–
GSM1234748	3T9.mycER.RNAseq.0hOHT_4	4/4	RNA-Seq	totRNA	mmu	3T9.mycER	–
GSM1234749	3T9.mycER.RNAseq.4hOHT_1	1/4	RNA-Seq	totRNA	mmu	3T9.mycER	GSM1234745-8
GSM1234750	3T9.mycER.RNAseq.4hOHT_2	2/4	RNA-Seq	totRNA	mmu	3T9.mycER	GSM1234745-8
GSM1234751	3T9.mycER.RNAseq.4hOHT_3	3/4	RNA-Seq	totRNA	mmu	3T9.mycER	GSM1234745-8
GSM1234752	3T9.mycER.RNAseq.4hOHT_4	4/4	RNA-Seq	totRNA	mmu	3T9.mycER	GSM1234745-8
GSM1234753	3T9.mycER.RNAseq.8hOHT_1	1/4	RNA-Seq	totRNA	mmu	3T9.mycER	GSM1234745-8
GSM1234754	3T9.mycER.RNAseq.8hOHT_2	2/4	RNA-Seq	totRNA	mmu	3T9.mycER	GSM1234745-8
GSM1234755	3T9.mycER.RNAseq.8hOHT_3	3/4	RNA-Seq	totRNA	mmu	3T9.mycER	GSM1234745-8
GSM1234756	3T9.mycER.RNAseq.8hOHT_4	4/4	RNA-Seq	totRNA	mmu	3T9.mycER	GSM1234745-8
GSM1234757	3T9.mycER.RNAseq.16hOHT_1	1/4	RNA-Seq	totRNA	mmu	3T9.mycER	GSM1234745-8
GSM1234758	3T9.mycER.RNAseq.16hOHT_2	2/4	RNA-Seq	totRNA	mmu	3T9.mycER	GSM1234745-8
GSM1234759	3T9.mycER.RNAseq.16hOHT_3	3/4	RNA-Seq	totRNA	mmu	3T9.mycER	GSM1234745-8
GSM1234760	3T9.mycER.RNAseq.16hOHT_4	4/4	RNA-Seq	totRNA	mmu	3T9.mycER	GSM1234745-8
GSM1234761	3T9.mycER.RNAseq.0hOHT_4sU	1/1	RNA-Seq	4sU-RNA	mmu	3T9.mycER	–
GSM1234762	3T9.mycER.RNAseq.4hOHT_4sU	1/1	RNA-Seq	4sU-RNA	mmu	3T9.mycER	GSM1234761
GSM1230377	3T9.mycER.DNAseI.0hOHT	1/2	DNAse-Seq	–	mmu	3T9.mycER	GSM1230379
GSM1395176	3T9.mycER.DNAseI.0hOHT.B	2/2	DNAse-Seq	–	mmu	3T9.mycER	GSM1230379
GSM1230378	3T9.mycER.DNAseI.4hOHT	1/2	DNAse-Seq	–	mmu	3T9.mycER	GSM1230379
GSM1395177	3T9.mycER.DNAseI.4hOHT.B	2/2	DNAse-Seq	–	mmu	3T9.mycER	GSM1230379
GSM1230379	3T9.mycER.input.DNAseI	1/1	DNAse-Seq	–	mmu	3T9.mycER	–
