# Supplementary material for: Selective transcriptional regulation by Myc: Experimental design and computational analysis of high-throughput sequencing data
Source: Data Brief. 2015 Feb 12;3:40–6. doi: 10.1016/j.dib.2015.02.003 (PMC4510069; doi:10.1016/j.dib.2015.02.003)
Supplement: Supplementary file 1 — Supplementary data [file mmc1.zip › README.rtf]

Source code used for the bioinformatics analyses in the article “Selective transcriptional regulation by Myc in cellular growth control and lymphomagenesis” by Sabò et al, Nature 2014.The archive saboEtAl2014_sourceCode.zip contains the following files:Software¥	compEpiTools_0.1.tar.gzAn R package (requires R_3.0.2) containing most of the functions and methods used, including R documentation and examples. The package is a preliminary version of a set of tools under submission to Bioconductor (manuscript in preparation).Environment settings¥	filemapping_GEO.RContains virtual links to the article NGS data (GSE51011) and processing steps to transform peaks list in GRanges R objects, which were saved in the file ./data/peaksRef.rda.¥	analysisEnvironment.RIs a script that is called at the beginning of the following analysis files to initialize the R session, including a number of additional compEpiTools functions and methods.¥	saboEtAl2014_Figures.RContains the bioinformatics code used to generate the article main figures.¥	saboEtAl2014_ExtData.RContains the bioinformatics code used to generate the article extended figures.¥	saboEtAl2014_ExtData10.RContains the bioinformatics code used to generate the article extended figure 10.Input and output¥	data directoryContains input and output R objects and text files (data).¥	figures directoryContains the figures resulting from the bioinformatic data analysis, which were used as panels to assemble the article main and extended figures.
