# Supplementary figures and images for: Selective transcriptional regulation by Myc: Experimental design and computational analysis of high-throughput sequencing data
Source: Data Brief. 2015 Feb 12;3:40–6. doi: 10.1016/j.dib.2015.02.003 (PMC4510069; doi:10.1016/j.dib.2015.02.003)

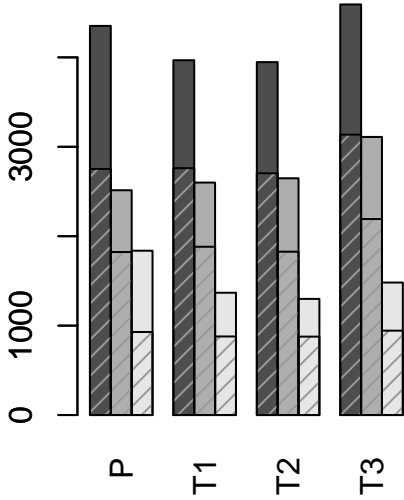

Supplement: Supplementary file 1 — Supplementary data [file mmc1.zip › figures/Fig2c.pdf]

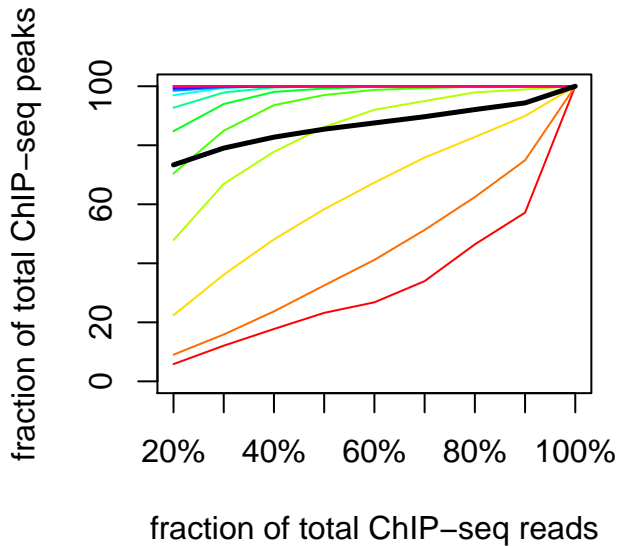

Supplement: Supplementary file 1 — Supplementary data [file mmc1.zip › figures/ExtDataFig1e.pdf]

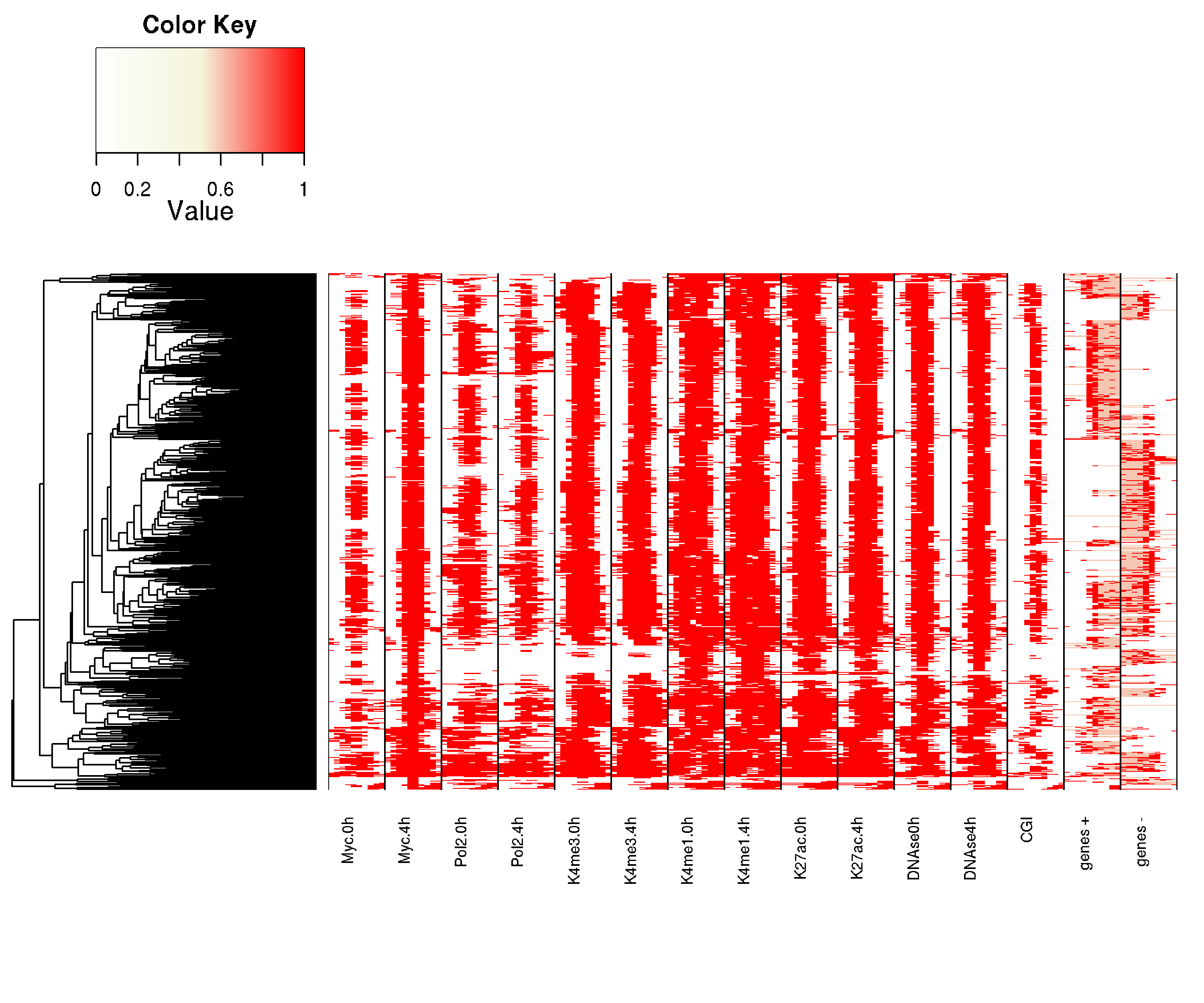

Supplement: Supplementary file 1 — Supplementary data [file mmc1.zip › figures/ExtDataFig7d_part1.png]

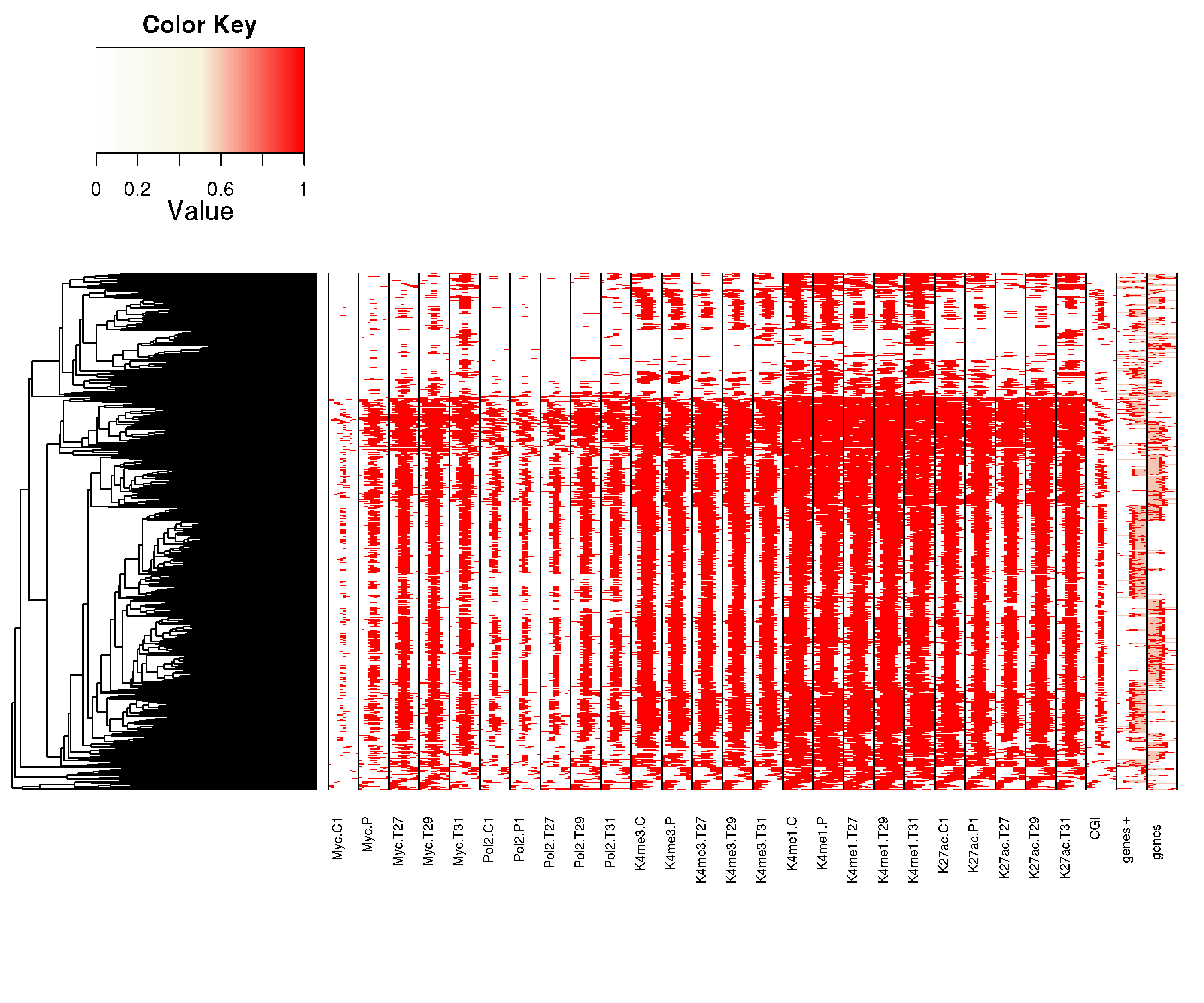

Supplement: Supplementary file 1 — Supplementary data [file mmc1.zip › figures/Fig1a.png]

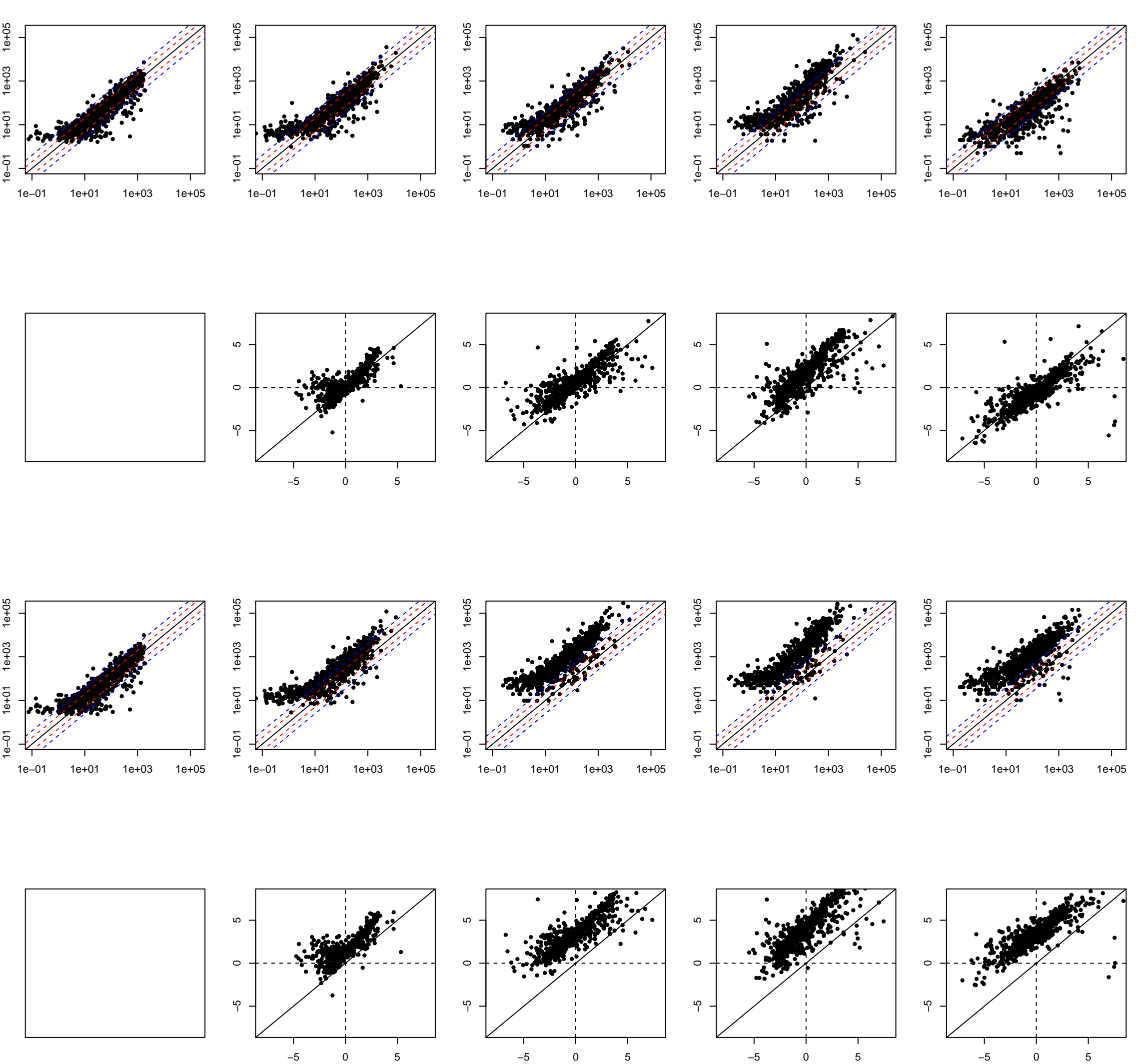

Supplement: Supplementary file 1 — Supplementary data [file mmc1.zip › figures/ExtDataFig5.pdf]

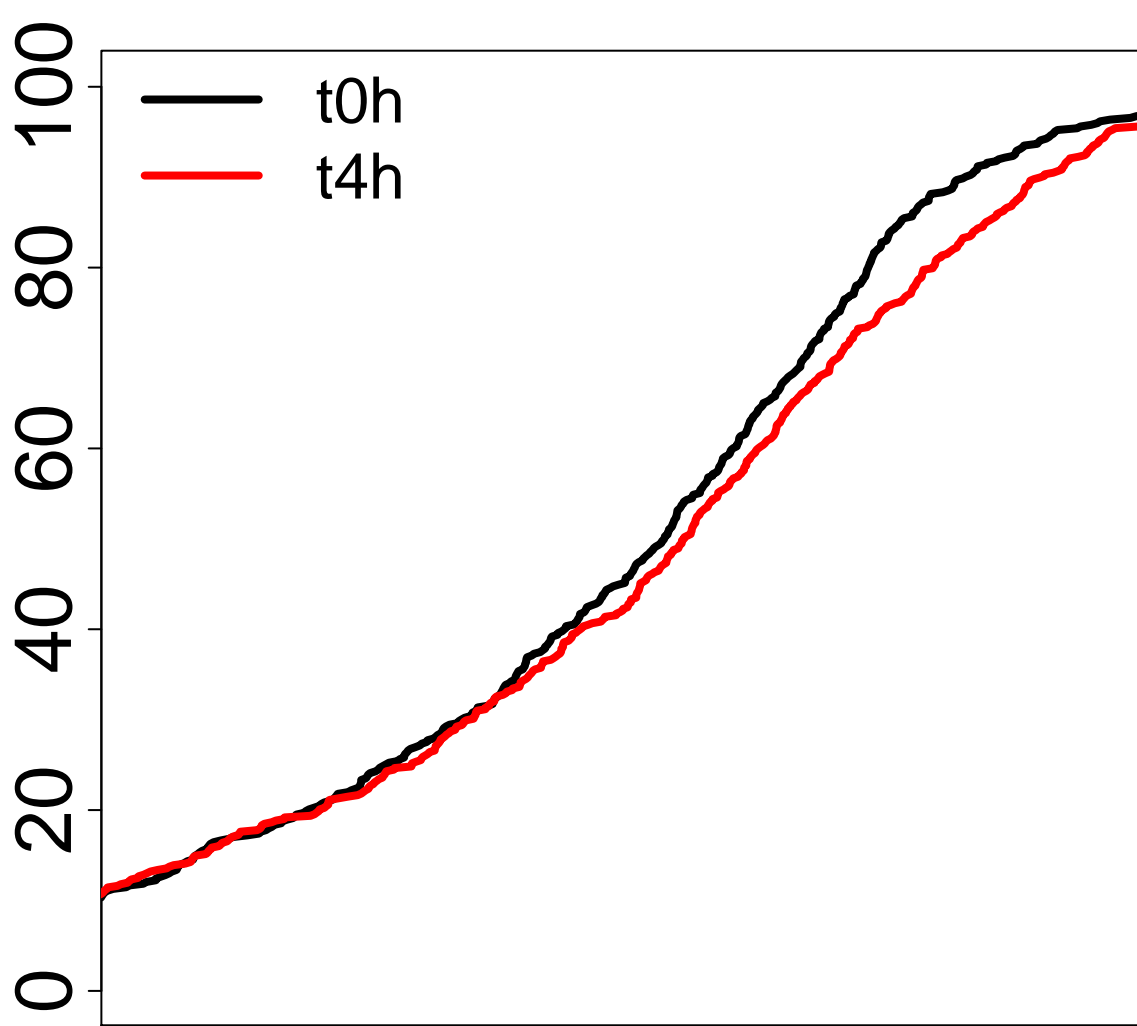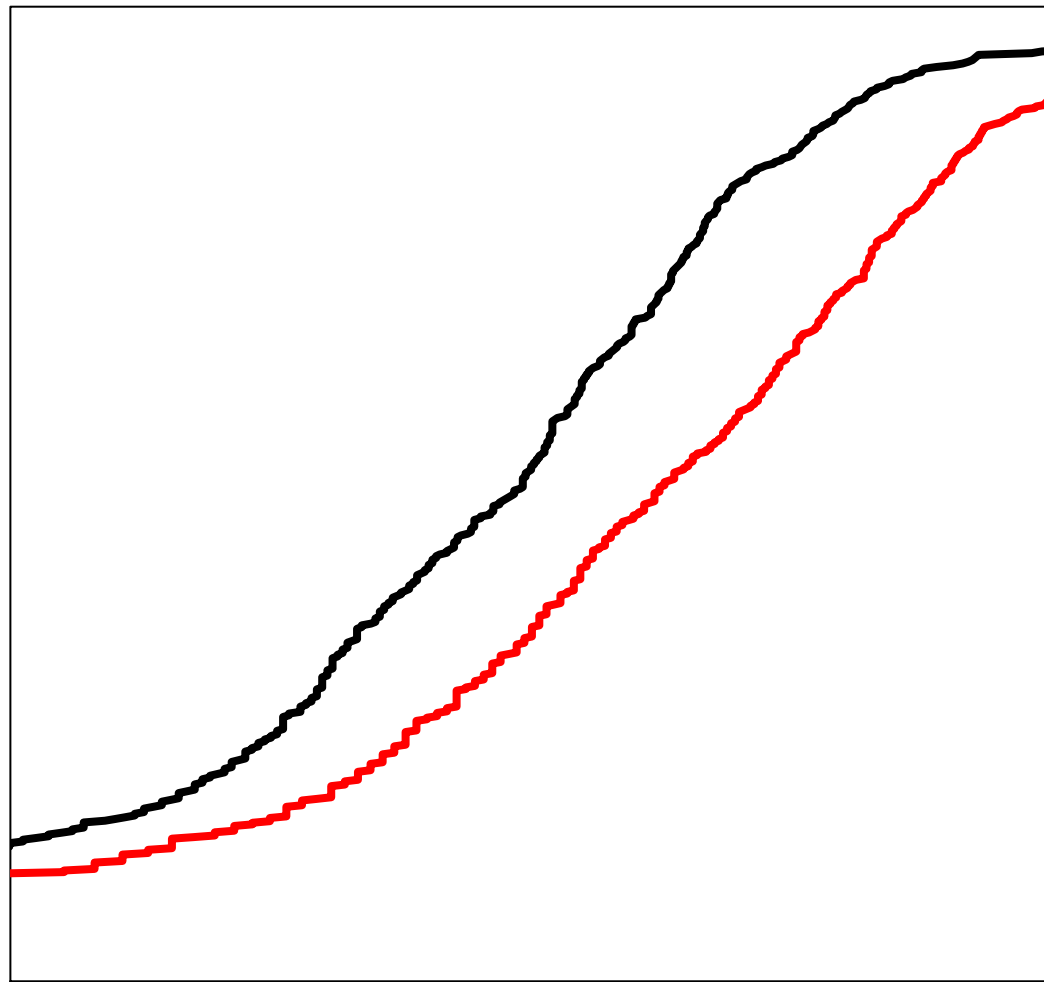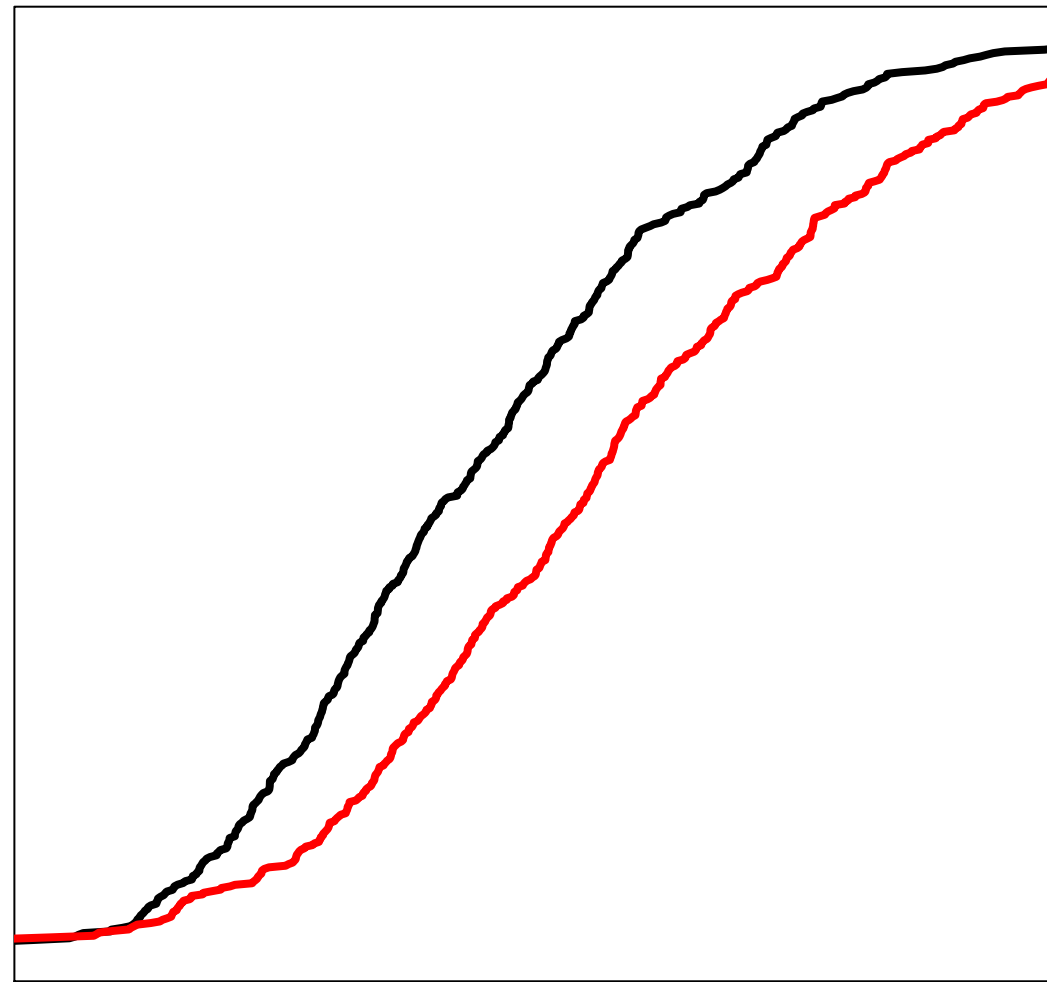

Supplement: Supplementary file 1 — Supplementary data [file mmc1.zip › figures/ExtDataFig10d.pdf]

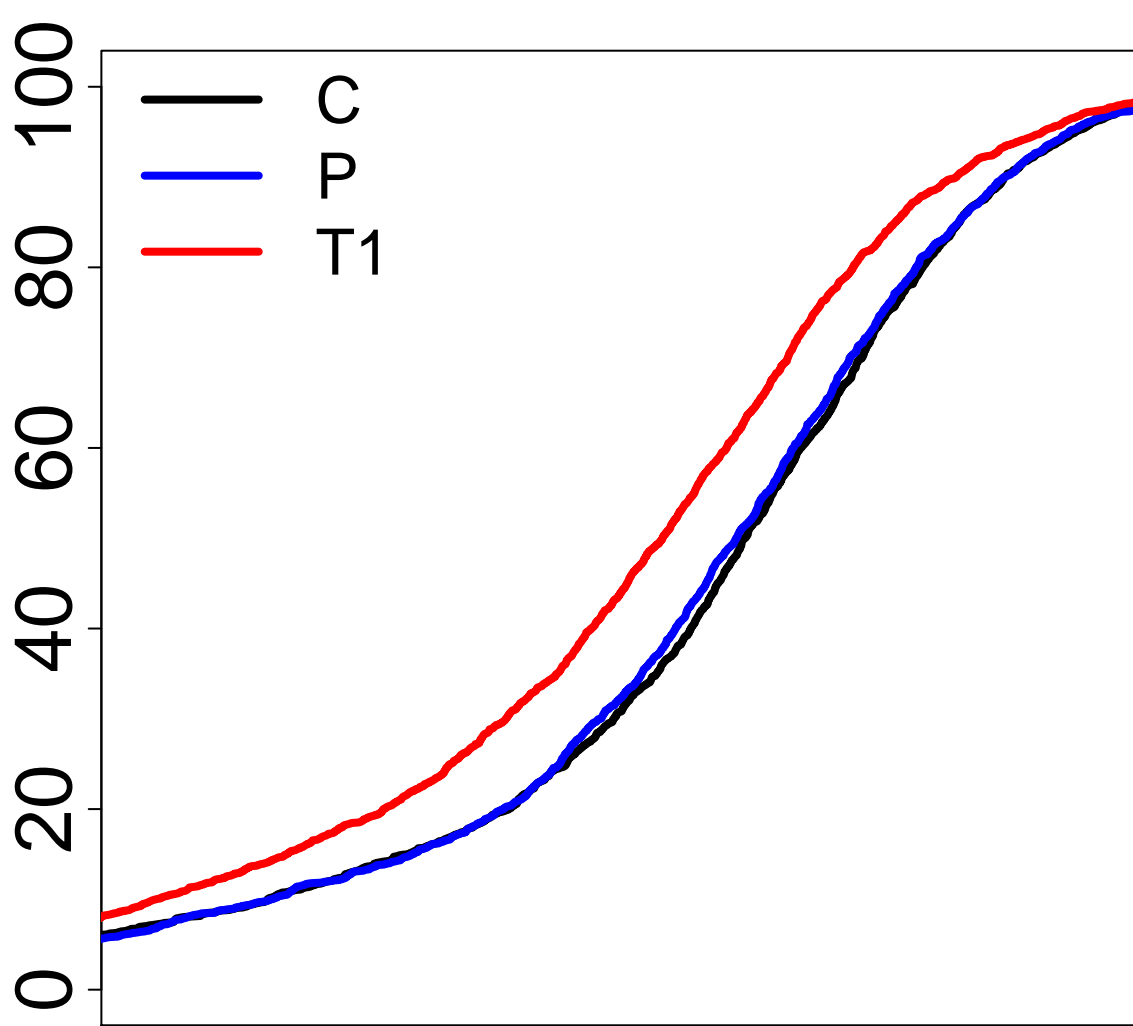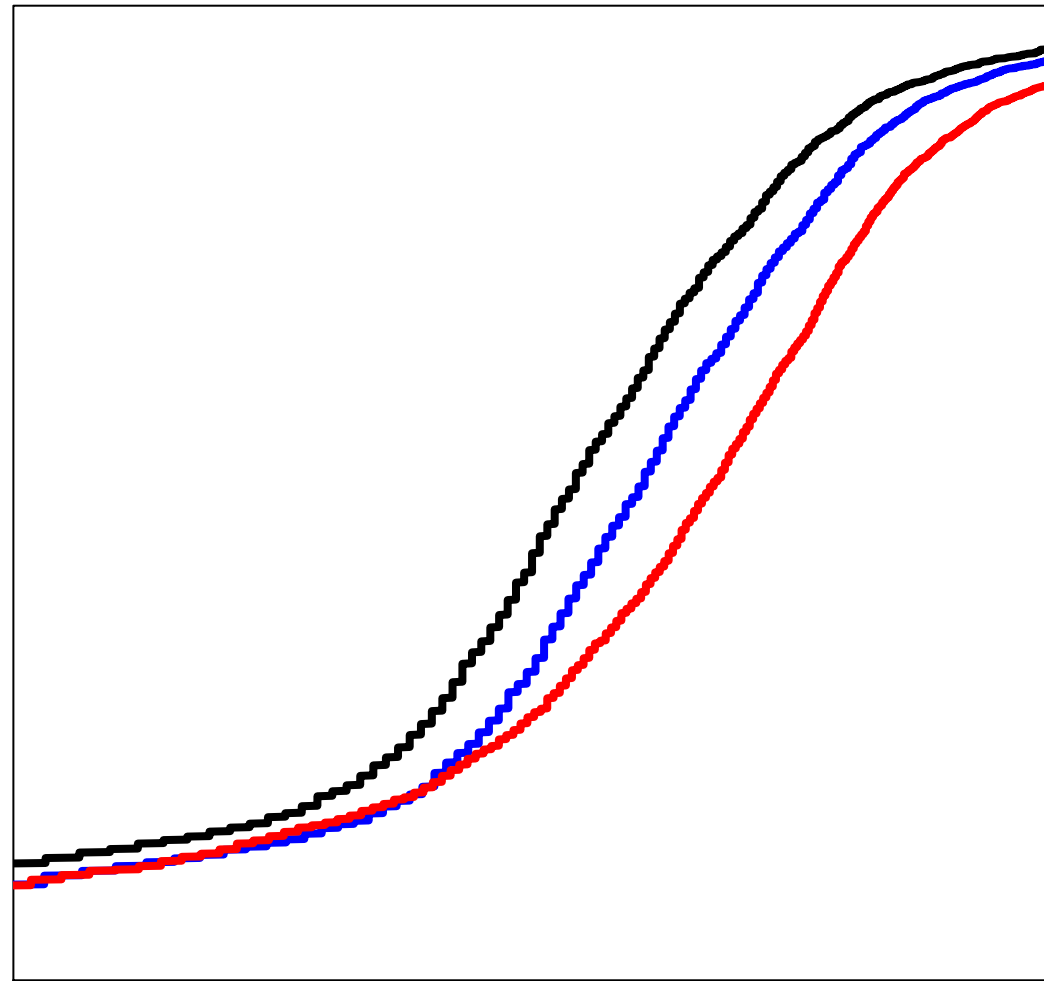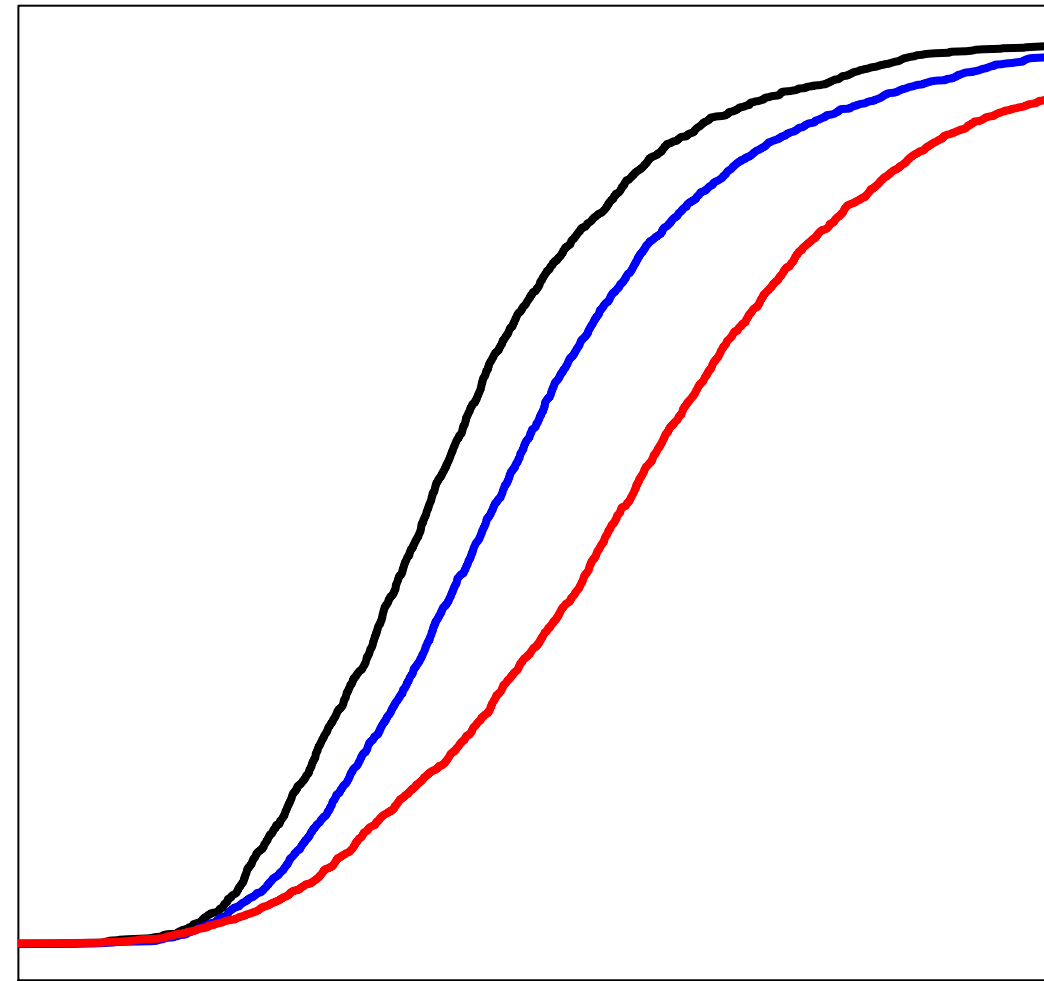

Supplement: Supplementary file 1 — Supplementary data [file mmc1.zip › figures/ExtDataFig10c.pdf]

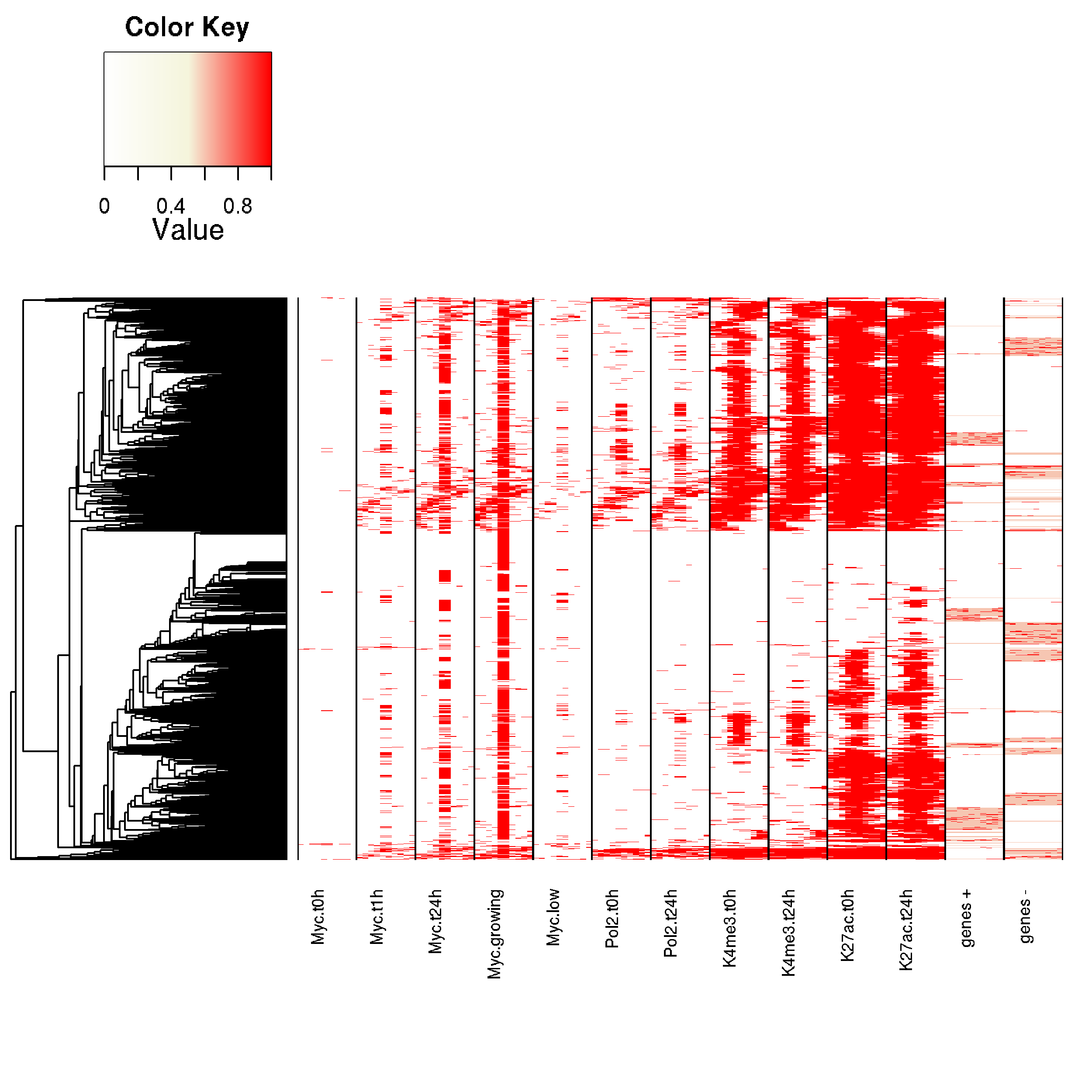

Supplement: Supplementary file 1 — Supplementary data [file mmc1.zip › figures/ExtDataFig6a_part2.png]

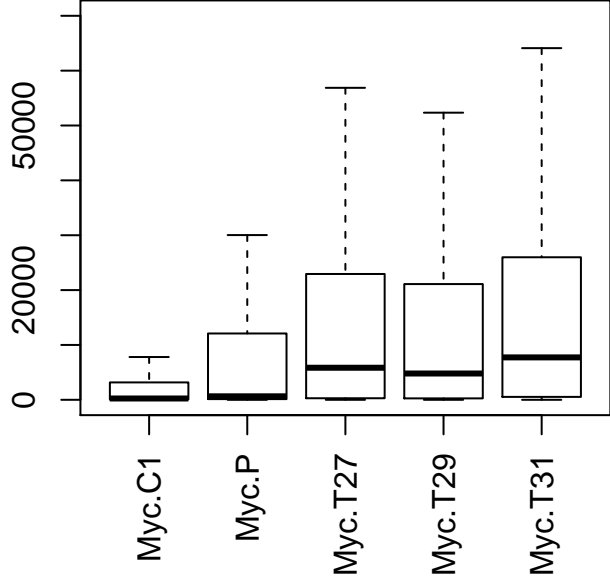

Supplement: Supplementary file 1 — Supplementary data [file mmc1.zip › figures/ExtDataFig1j.pdf]

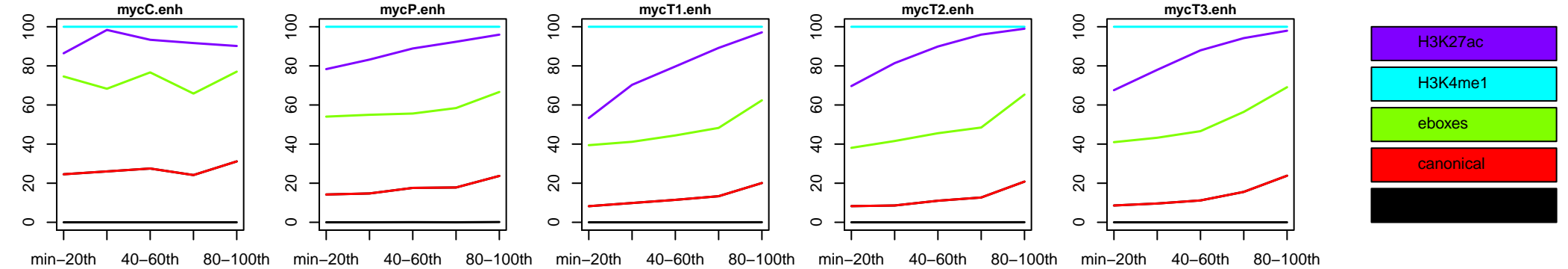

Supplement: Supplementary file 1 — Supplementary data [file mmc1.zip › figures/ExtDataFig8b_part2.pdf]

## Color Key

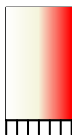

0 250  
Value

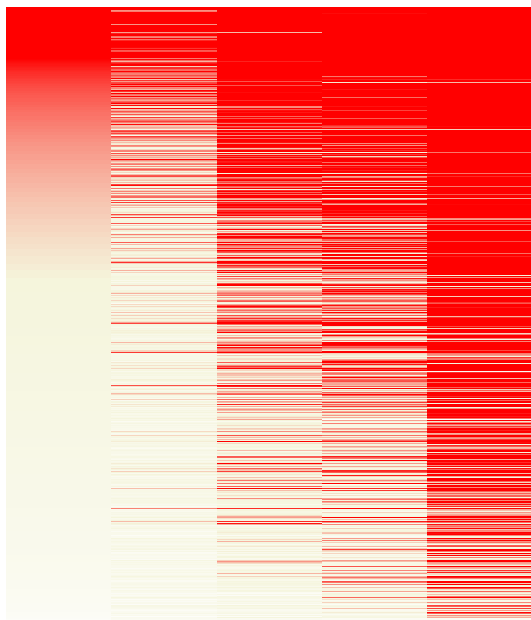

SD

SA 1h

SA 2h

OHT 0h

OHT 4h

Supplement: Supplementary file 1 — Supplementary data [file mmc1.zip › figures/ExtDataFig8a_part2.pdf]

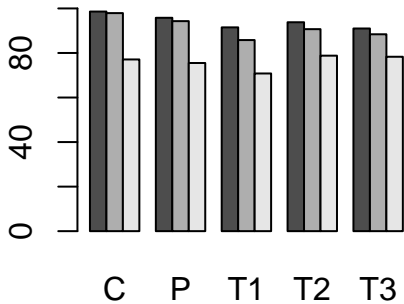

Supplement: Supplementary file 1 — Supplementary data [file mmc1.zip › figures/ExtDataFig2a.pdf]

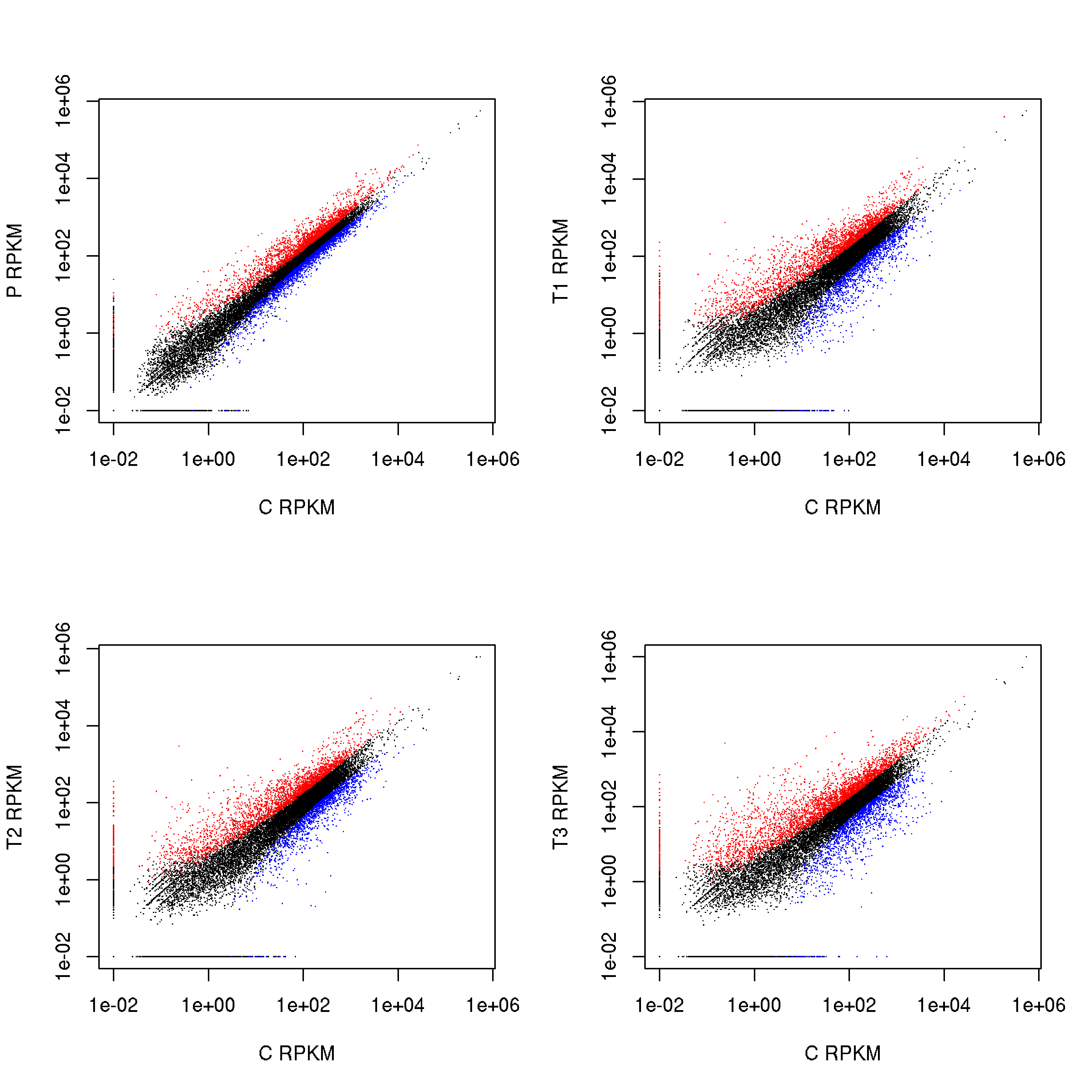

Supplement: Supplementary file 1 — Supplementary data [file mmc1.zip › figures/ExtDataFig4b.png]

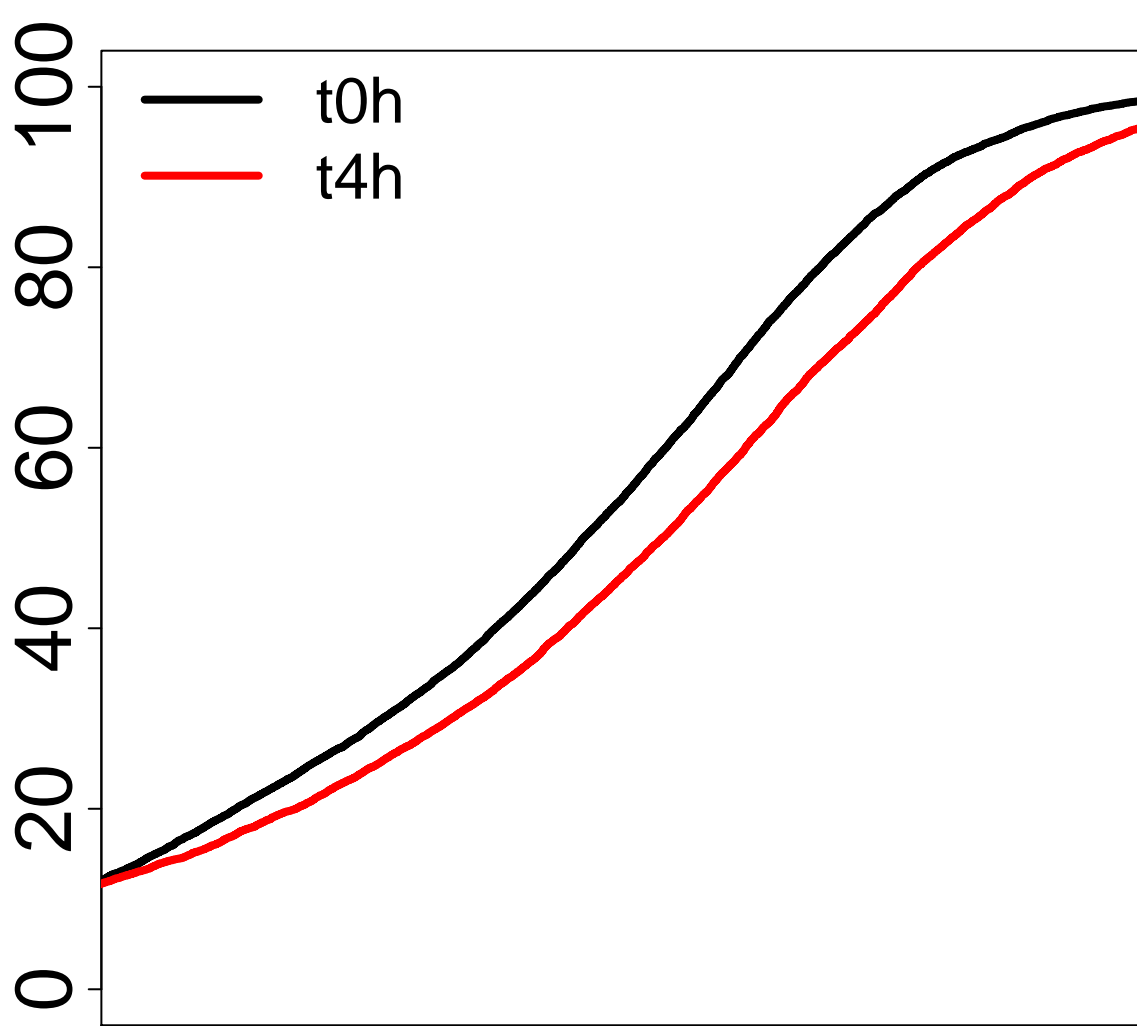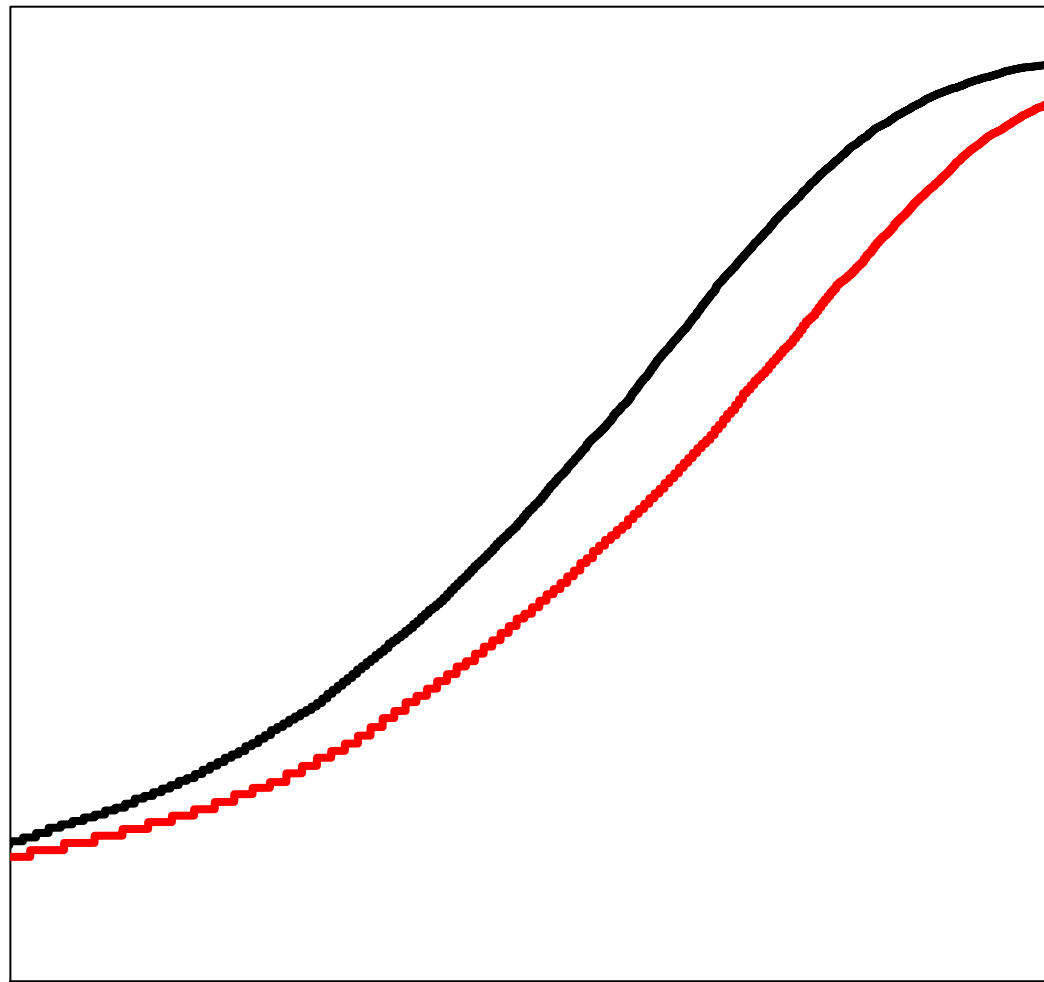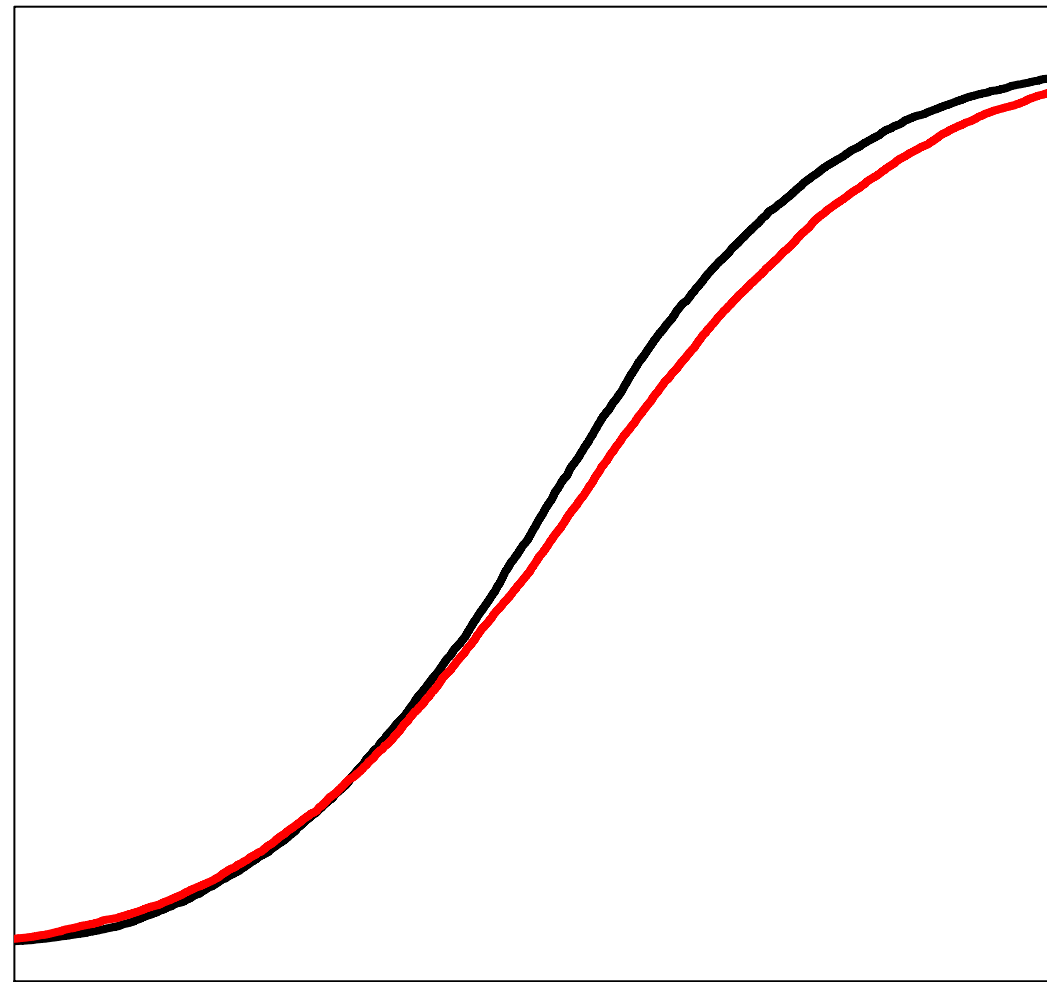

Supplement: Supplementary file 1 — Supplementary data [file mmc1.zip › figures/ExtDataFig10b.pdf]

**C**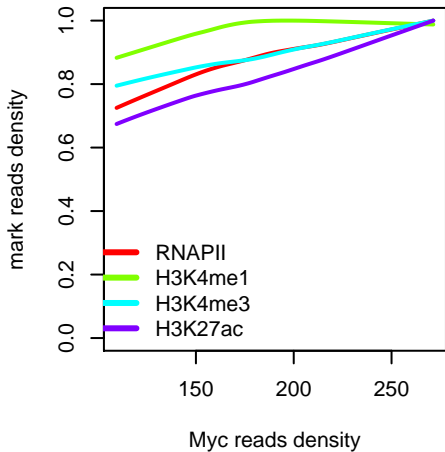**P**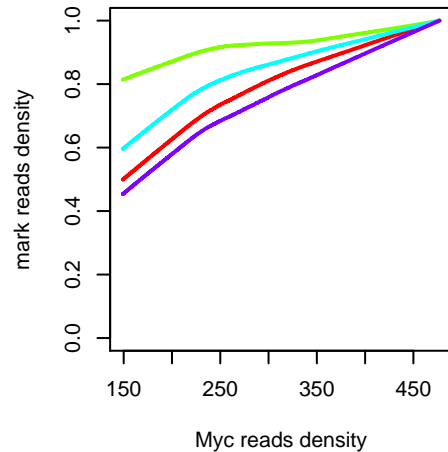**T1**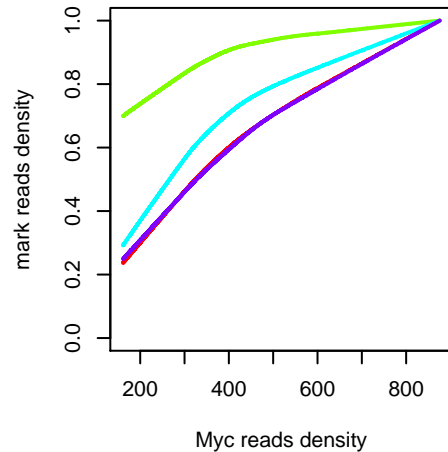**T2**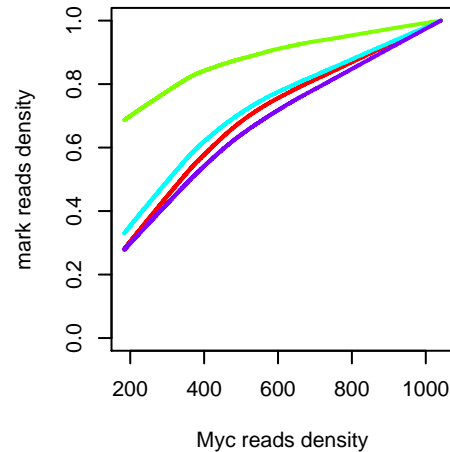**T3**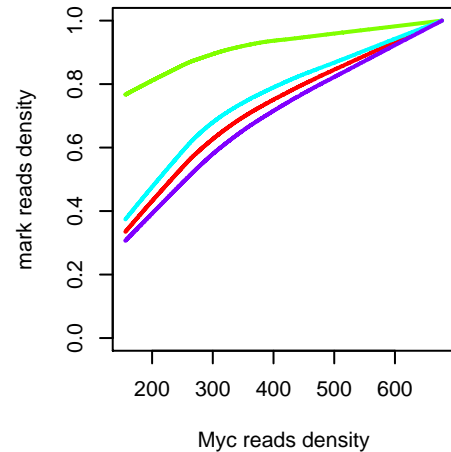

Supplement: Supplementary file 1 — Supplementary data [file mmc1.zip › figures/ExtDataFig2d_part1.pdf]

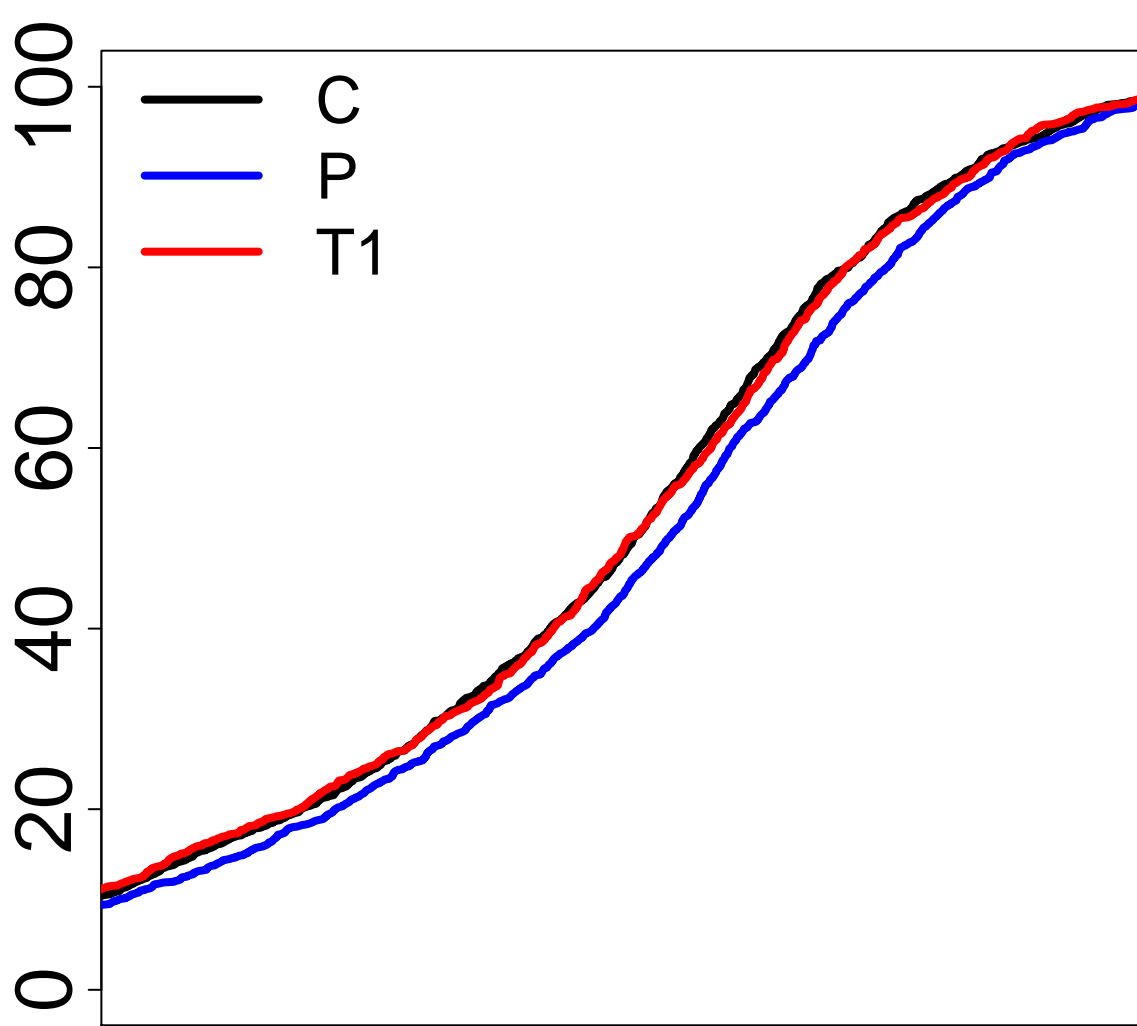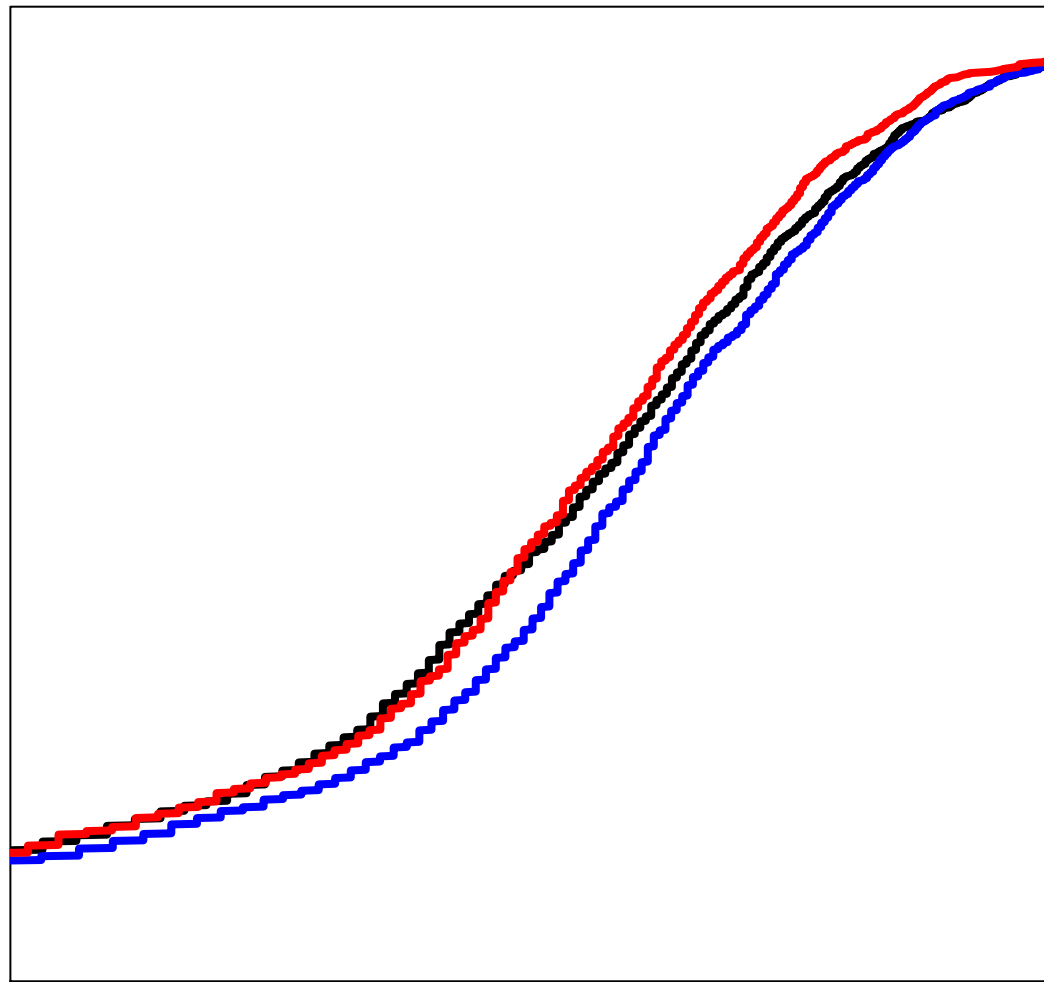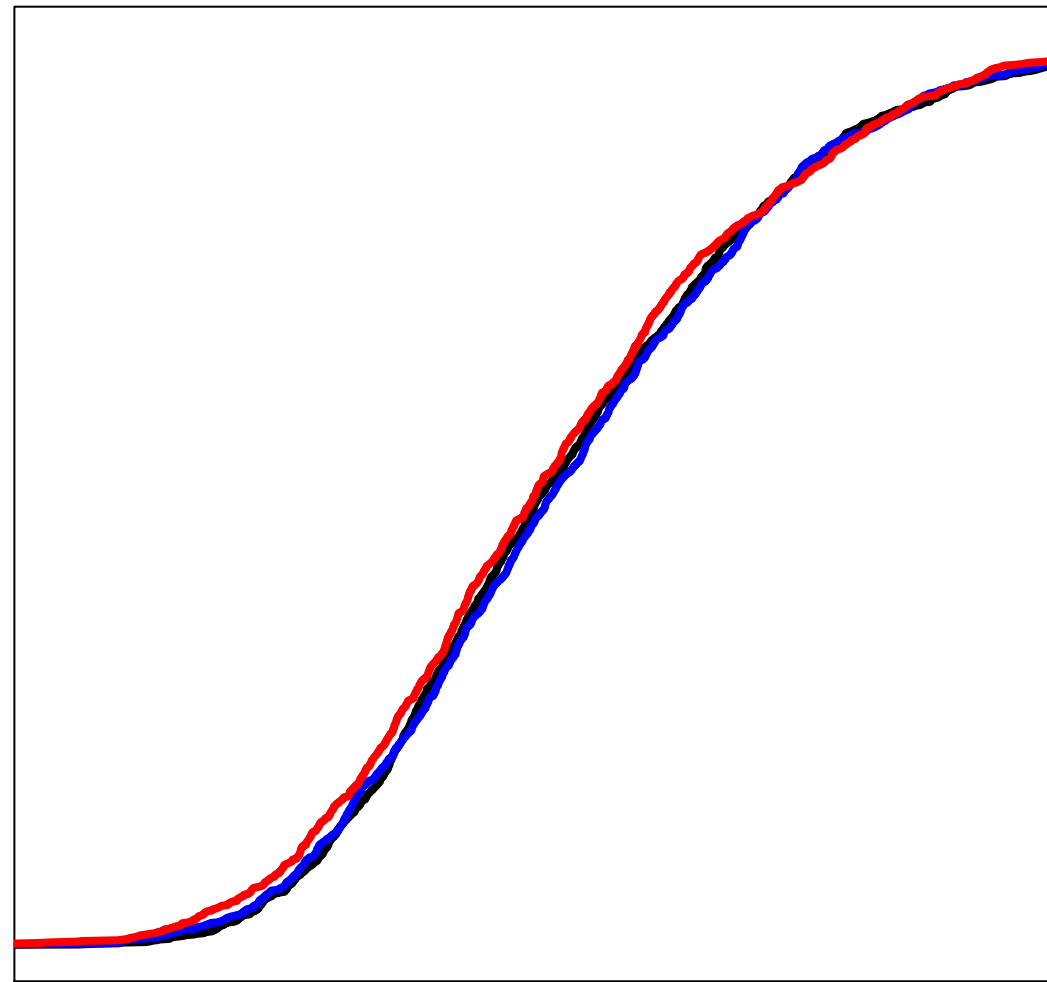

Supplement: Supplementary file 1 — Supplementary data [file mmc1.zip › figures/ExtDataFig10e.pdf]

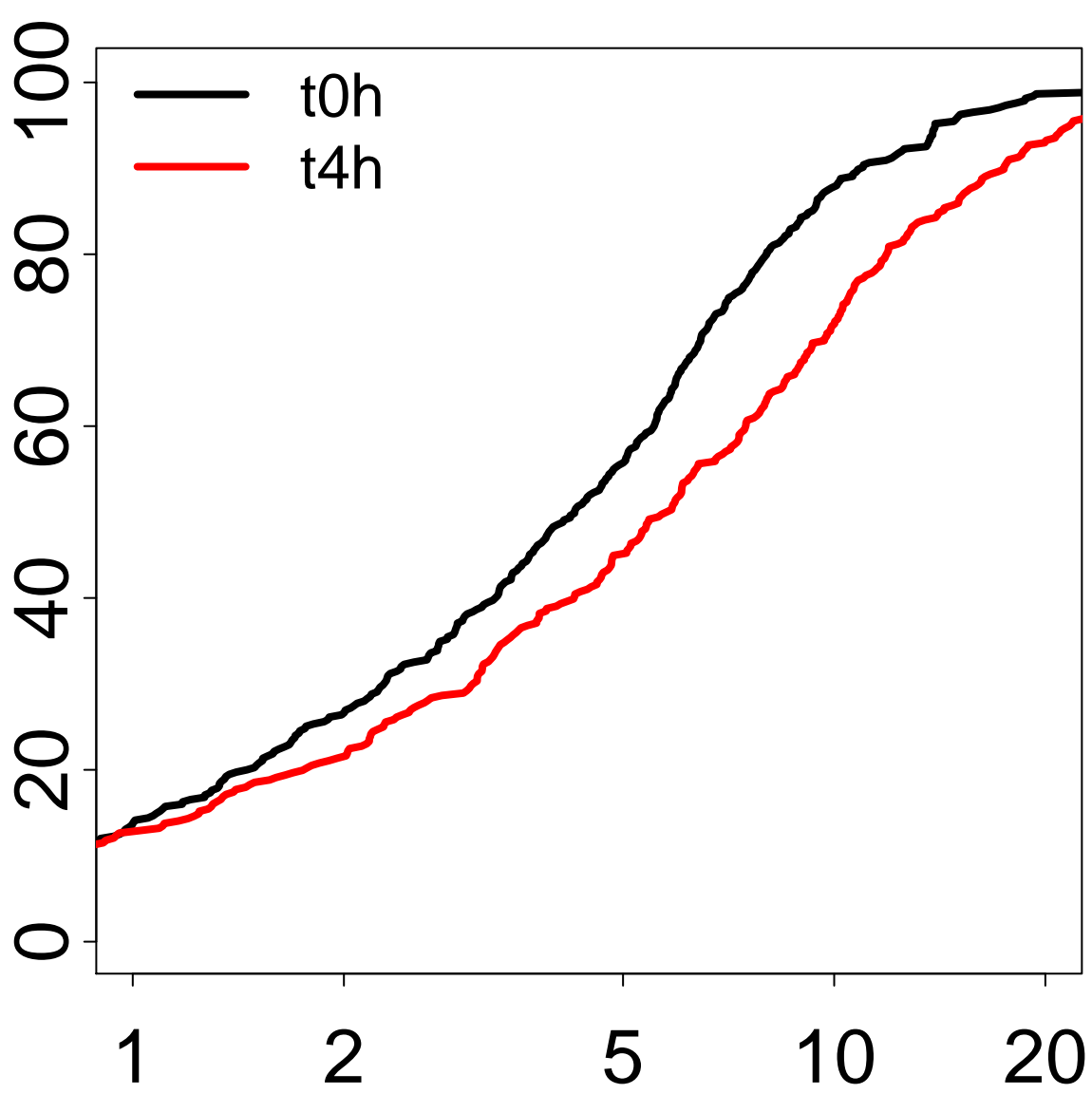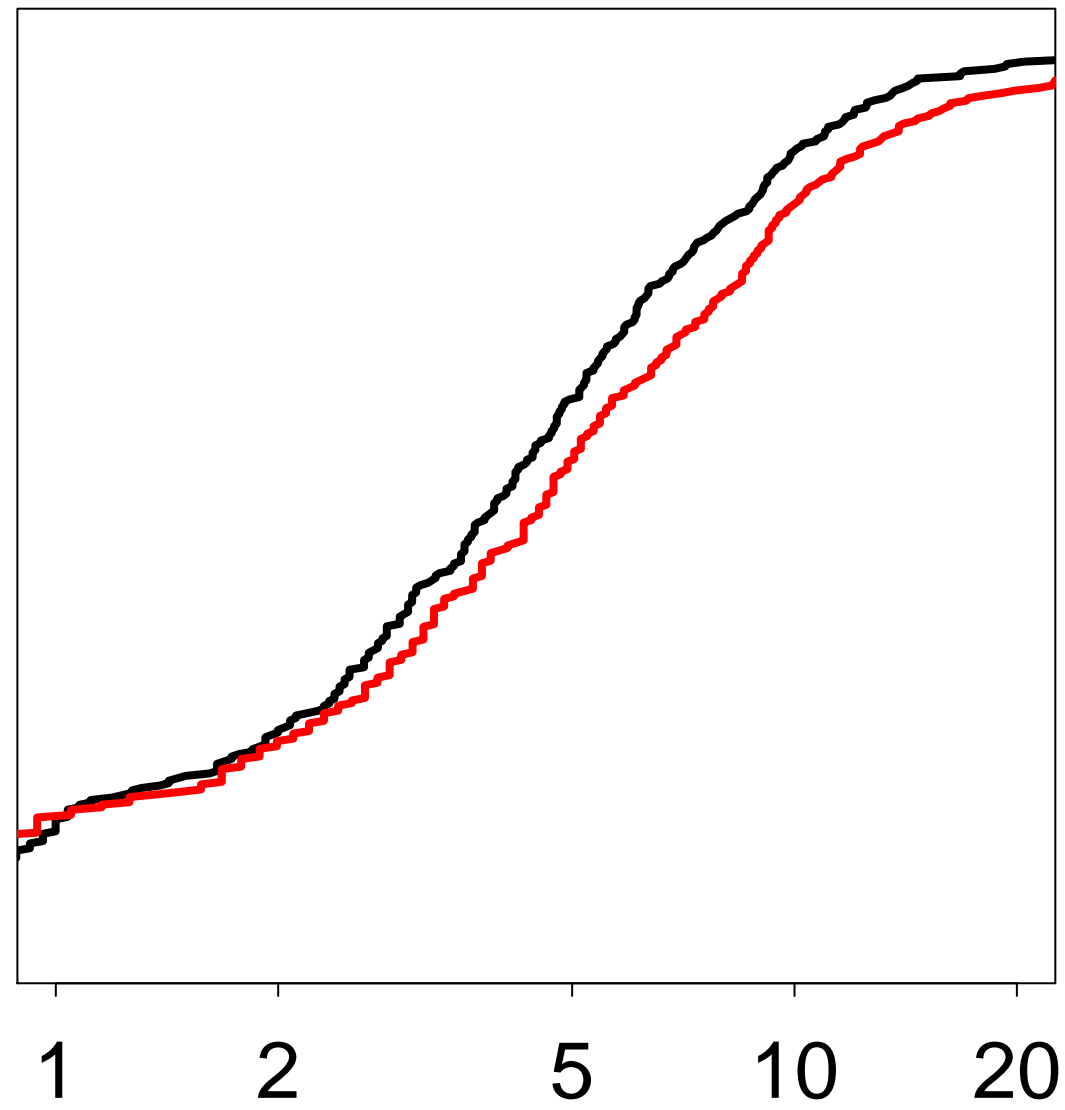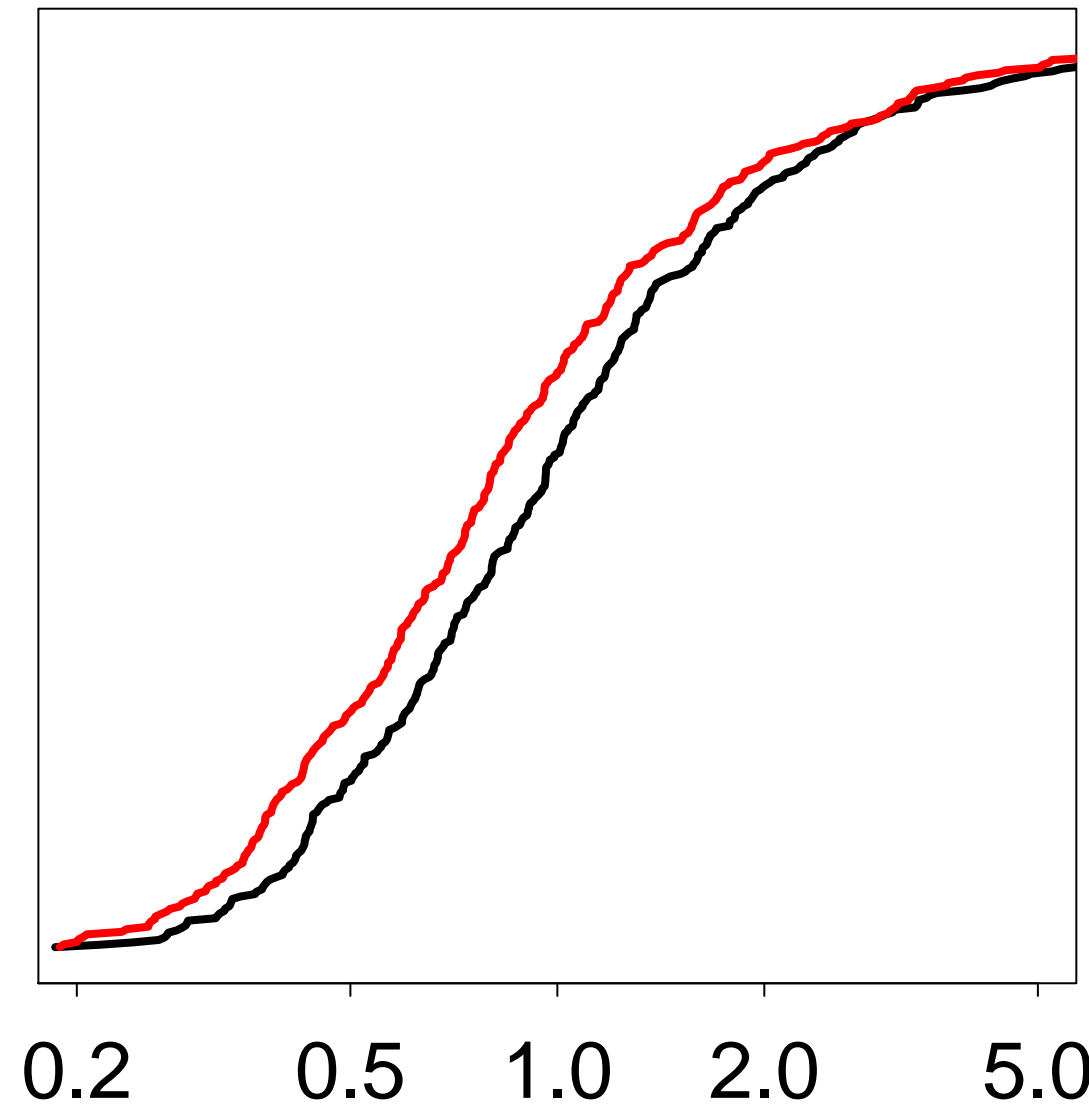

Supplement: Supplementary file 1 — Supplementary data [file mmc1.zip › figures/ExtDataFig10f.pdf]

**C**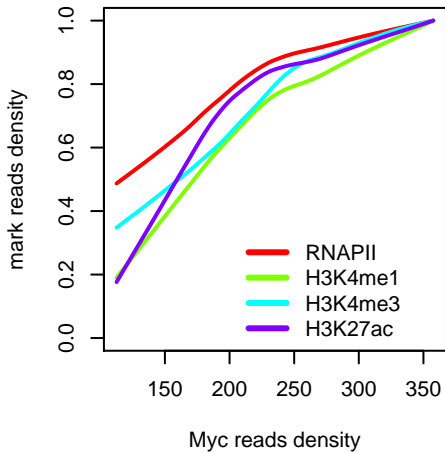**P**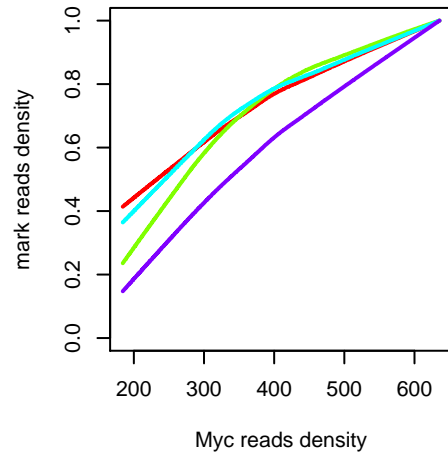**T1**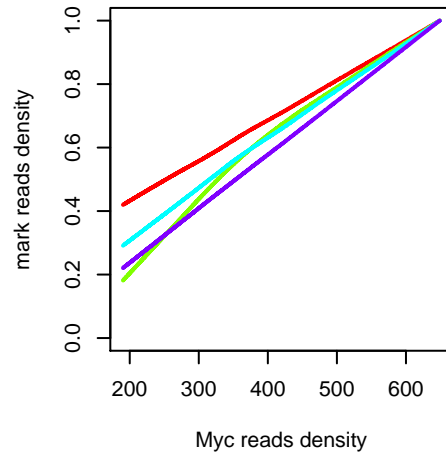**T2**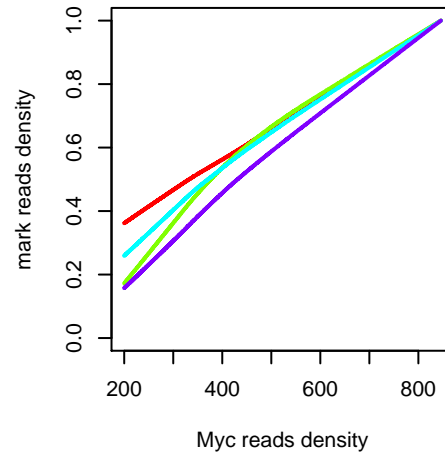**T3**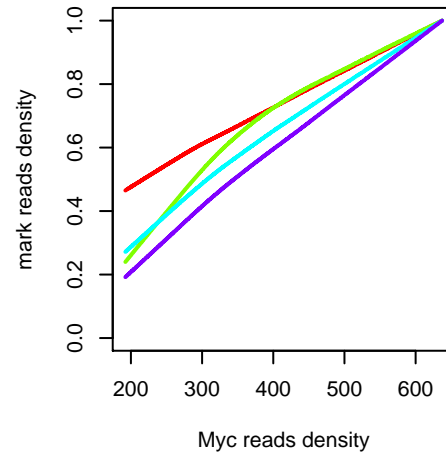

Supplement: Supplementary file 1 — Supplementary data [file mmc1.zip › figures/ExtDataFig2d_part2.pdf]

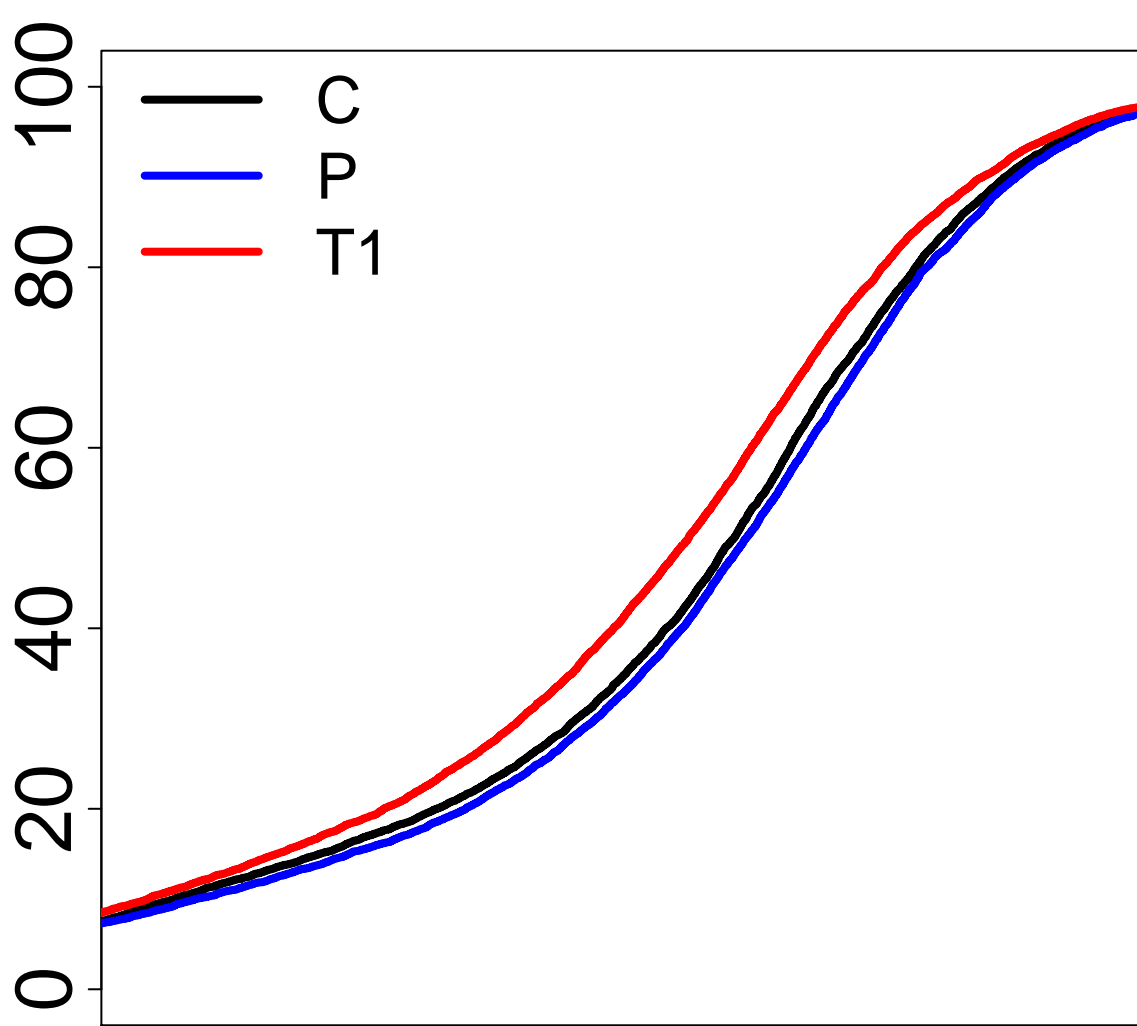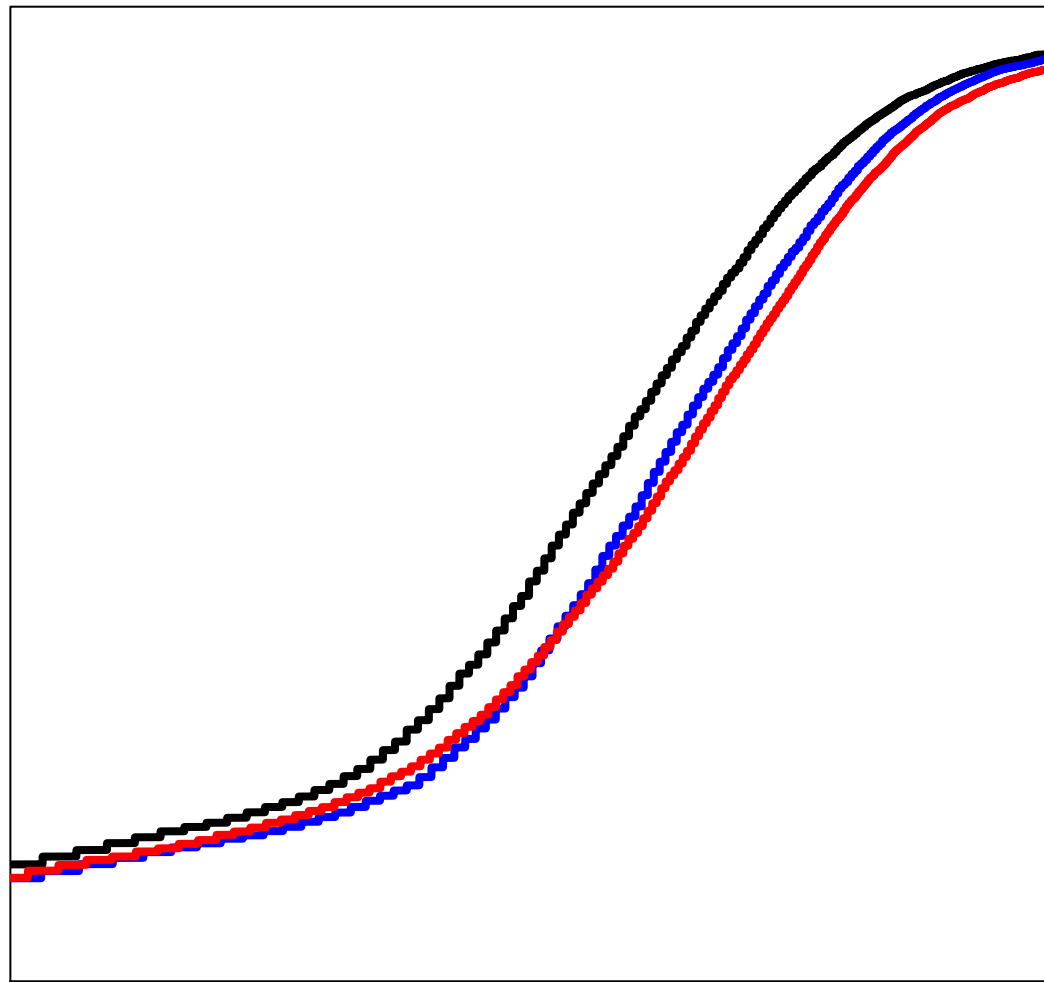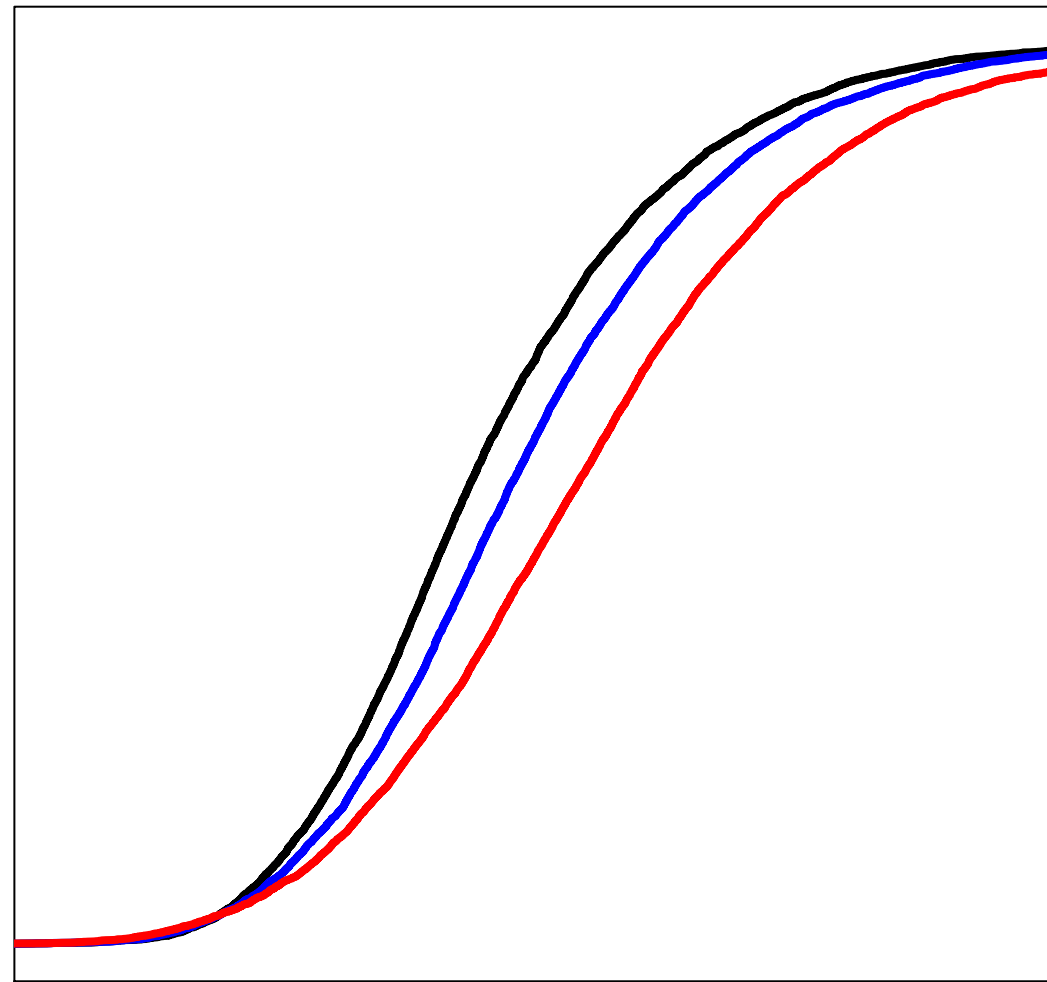

Supplement: Supplementary file 1 — Supplementary data [file mmc1.zip › figures/ExtDataFig10a.pdf]

# Cluster Dendrogram

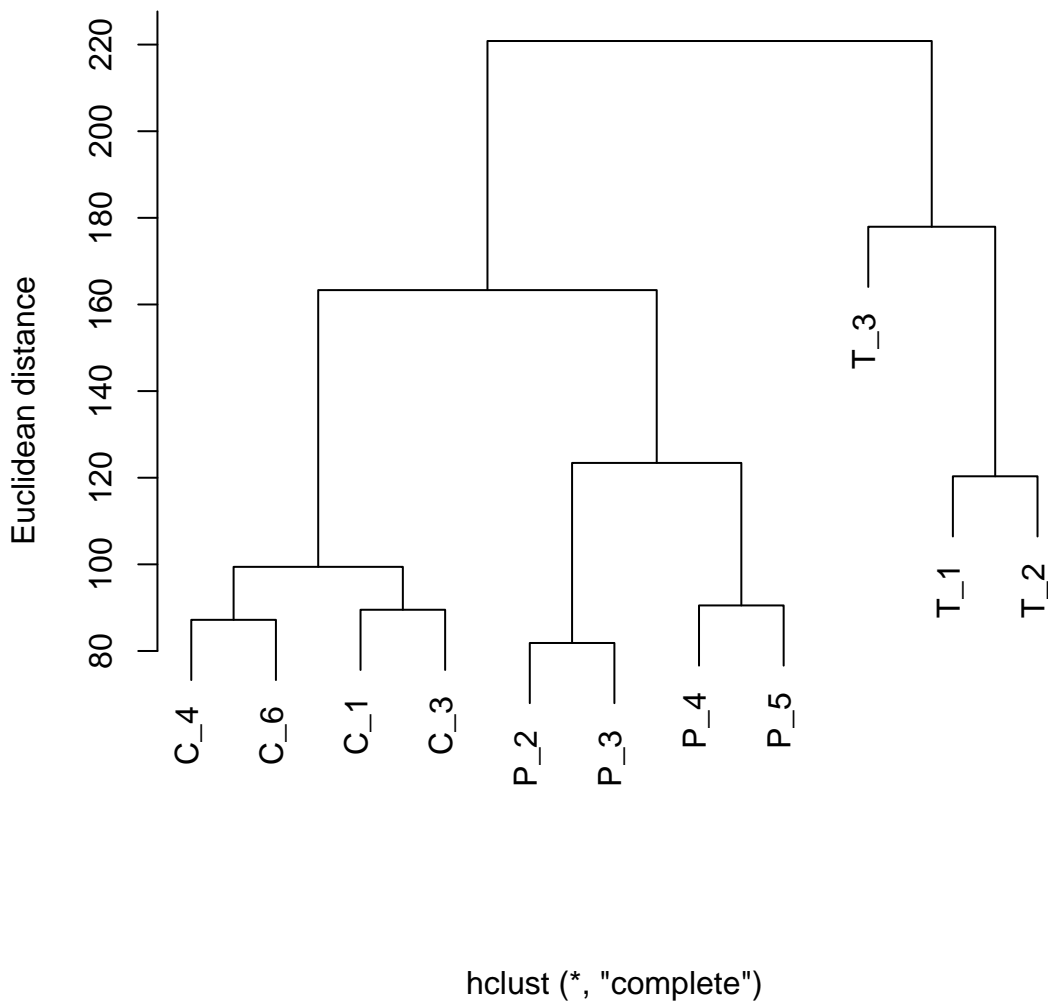

Supplement: Supplementary file 1 — Supplementary data [file mmc1.zip › figures/ExtDataFig4a.pdf]

Color Key

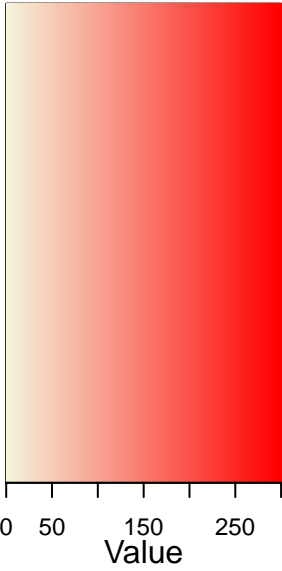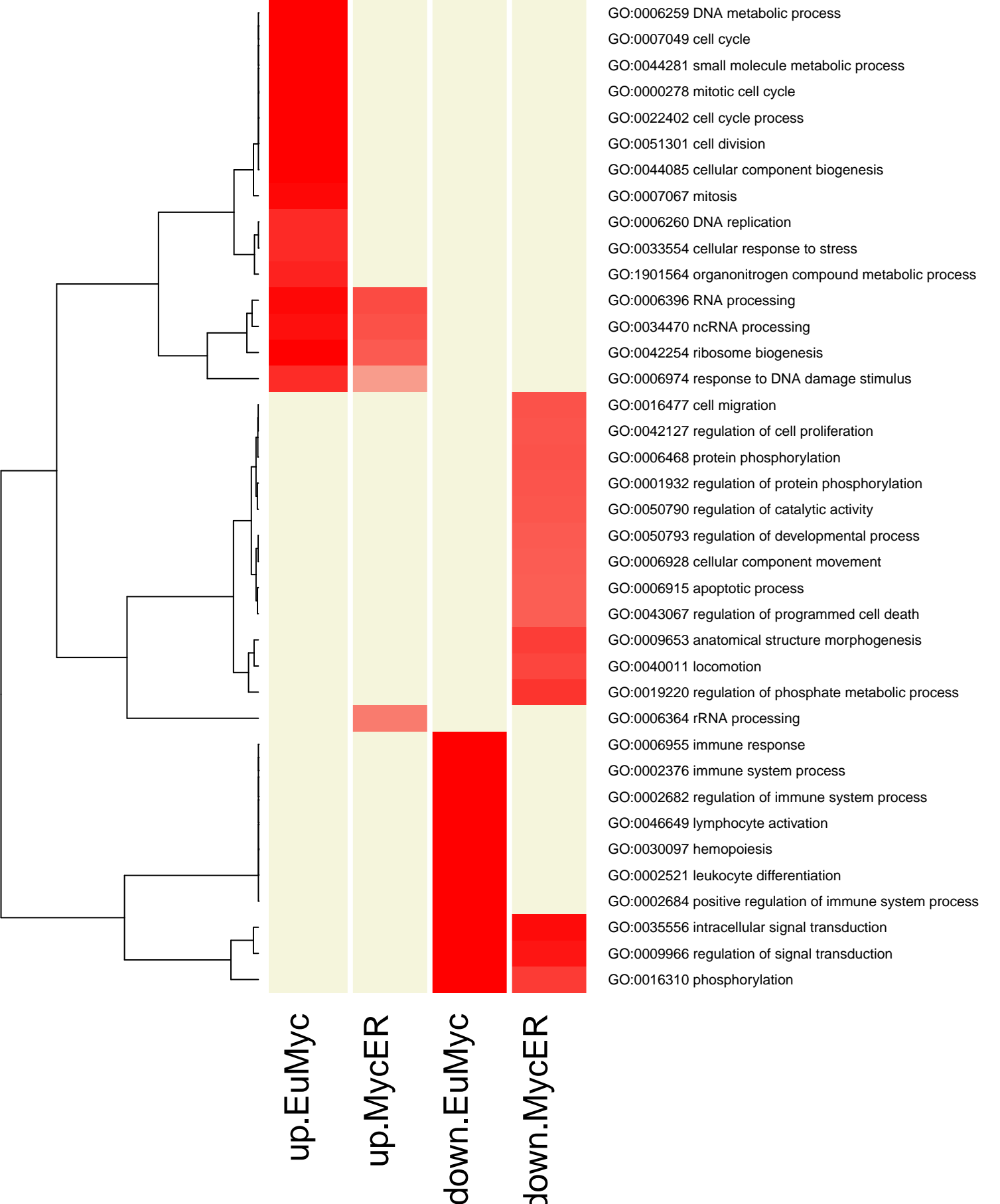

Supplement: Supplementary file 1 — Supplementary data [file mmc1.zip › figures/ExtDataFig9.pdf]

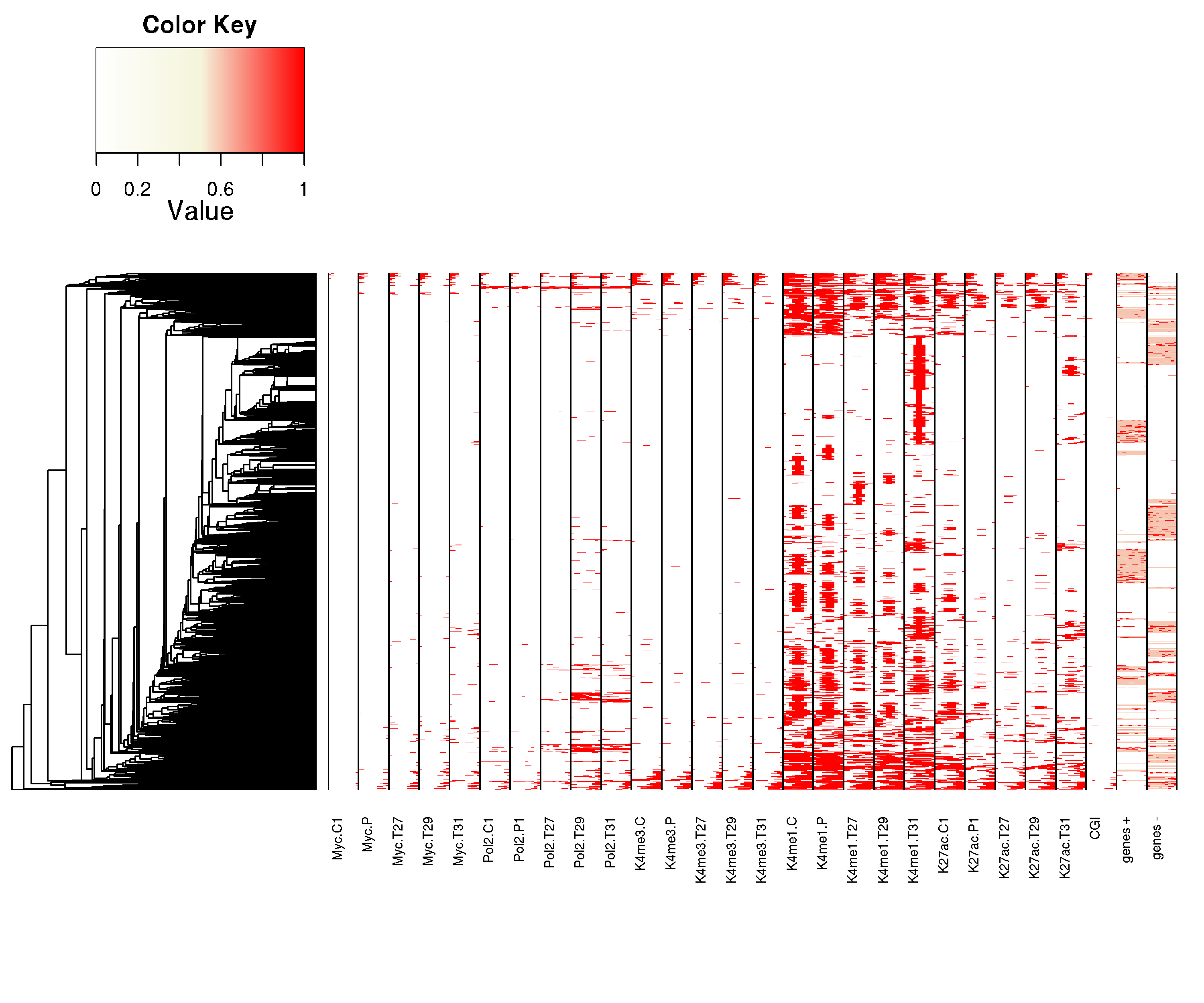

Supplement: Supplementary file 1 — Supplementary data [file mmc1.zip › figures/Fig1d.png]

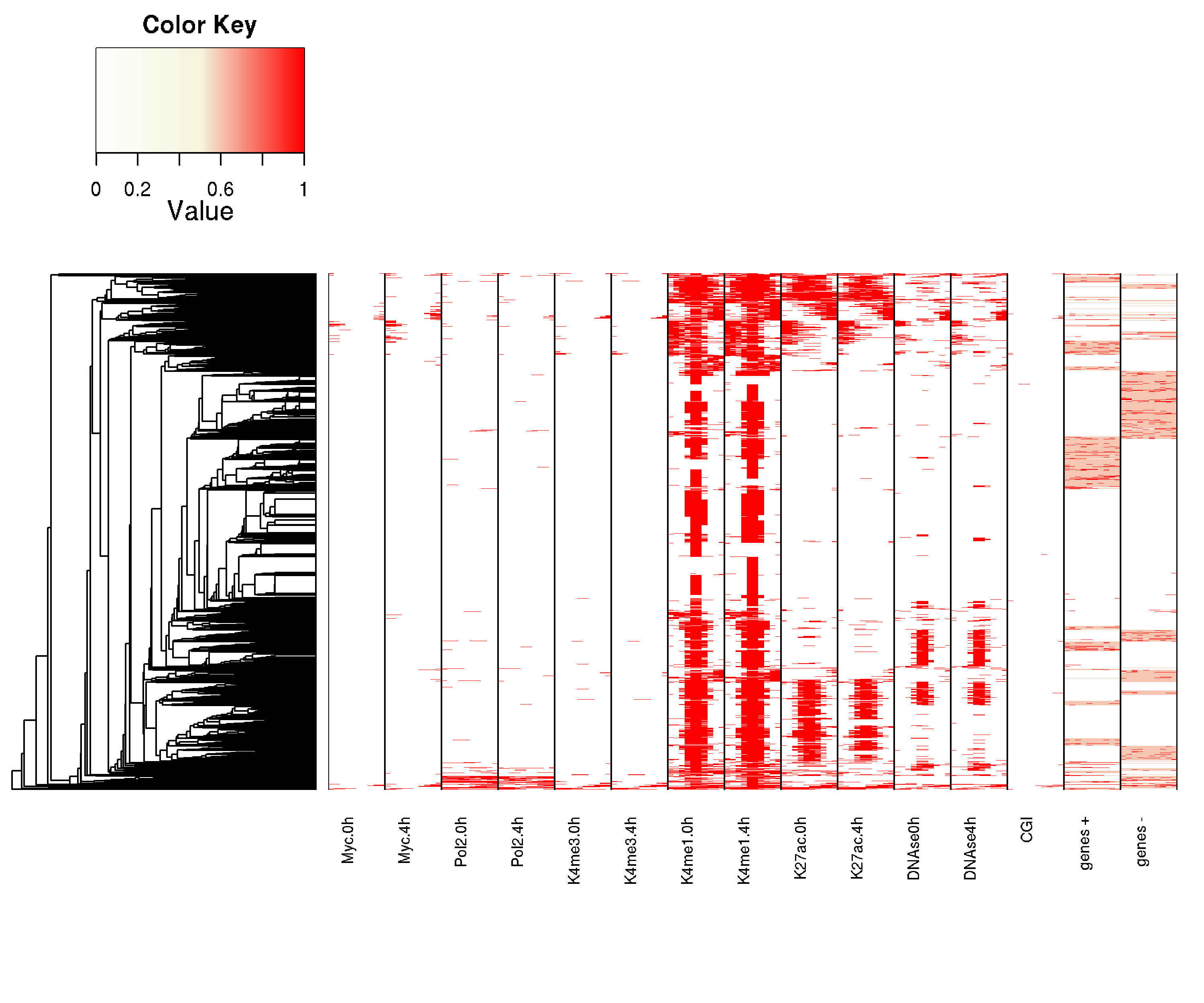

Supplement: Supplementary file 1 — Supplementary data [file mmc1.zip › figures/ExtDataFig7d_part4.png]

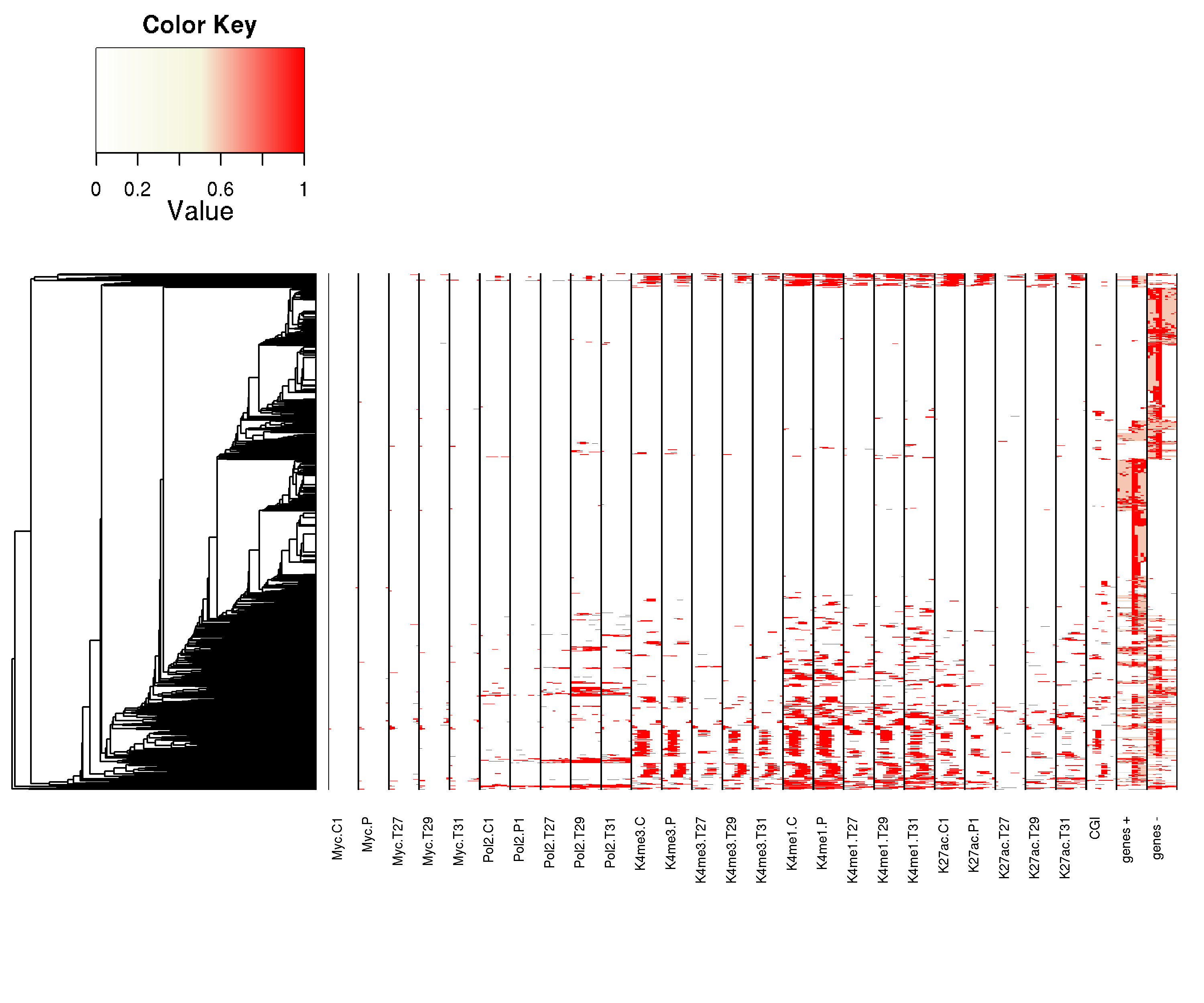

Supplement: Supplementary file 1 — Supplementary data [file mmc1.zip › figures/Fig1c.png]

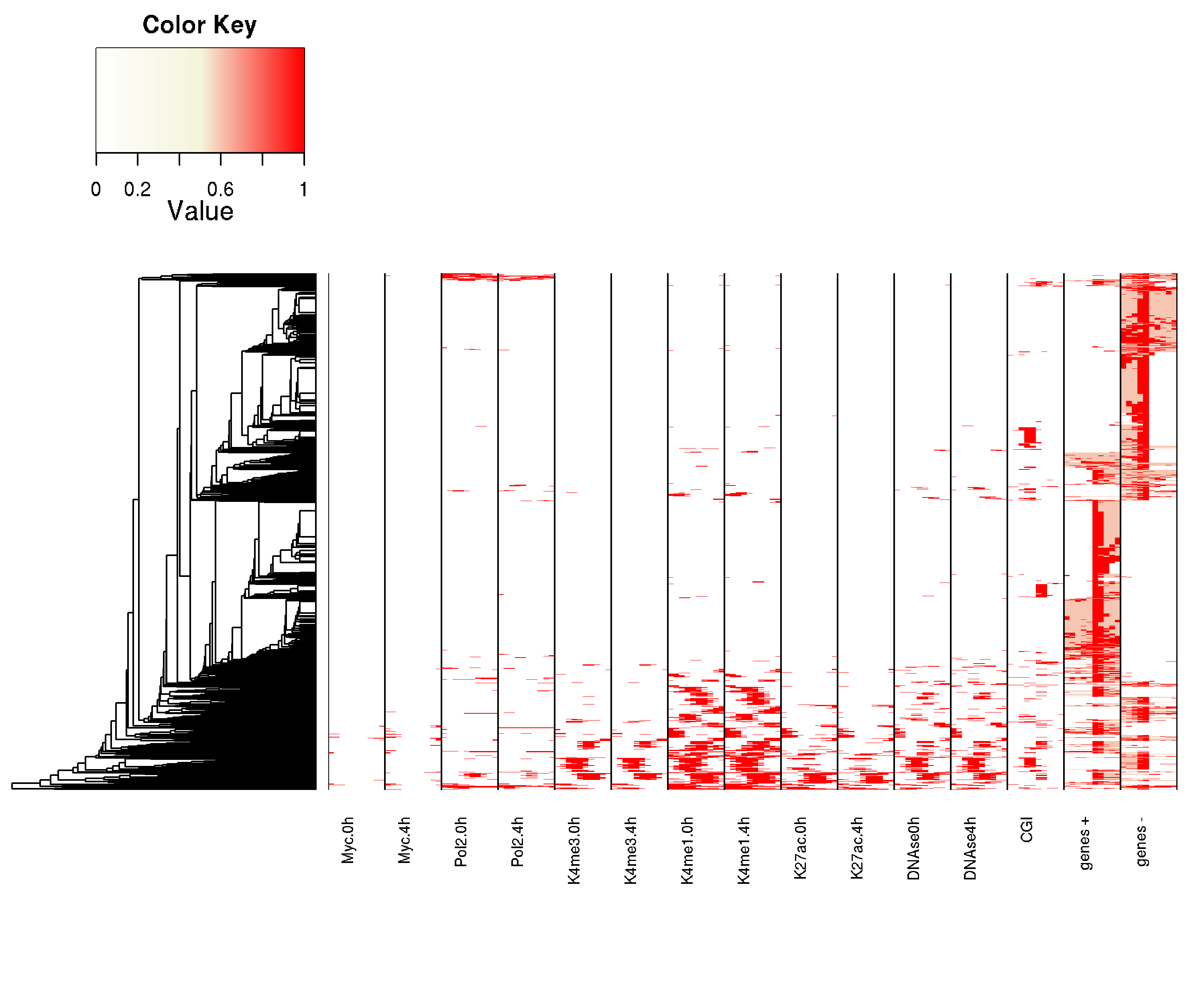

Supplement: Supplementary file 1 — Supplementary data [file mmc1.zip › figures/ExtDataFig7d_part3.png]

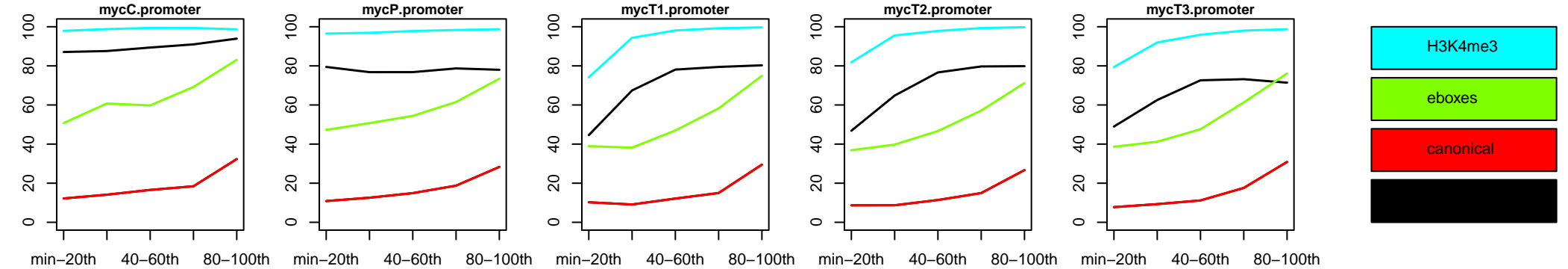

Supplement: Supplementary file 1 — Supplementary data [file mmc1.zip › figures/ExtDataFig8b_part1.pdf]

## Color Key

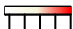

20 100  
Value

|         |     |     |     |     |     |         |
|---------|-----|-----|-----|-----|-----|---------|
|         | 100 | 94  | 86  | 86  | 84  | Myc.C1  |
|         | 33  | 100 | 85  | 84  | 77  | Myc.P   |
|         | 17  | 48  | 100 | 75  | 64  | Myc.T27 |
|         | 19  | 51  | 81  | 100 | 66  | Myc.T29 |
|         | 15  | 37  | 56  | 53  | 100 | Myc.T31 |
| Myc.C1  |     |     |     |     |     |         |
| Myc.P   |     |     |     |     |     |         |
| Myc.T27 |     |     |     |     |     |         |
| Myc.T29 |     |     |     |     |     |         |
| Myc.T31 |     |     |     |     |     |         |

Supplement: Supplementary file 1 — Supplementary data [file mmc1.zip › figures/ExtDataFig1i.pdf]

## Color Key

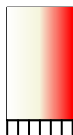

0 250  
Value

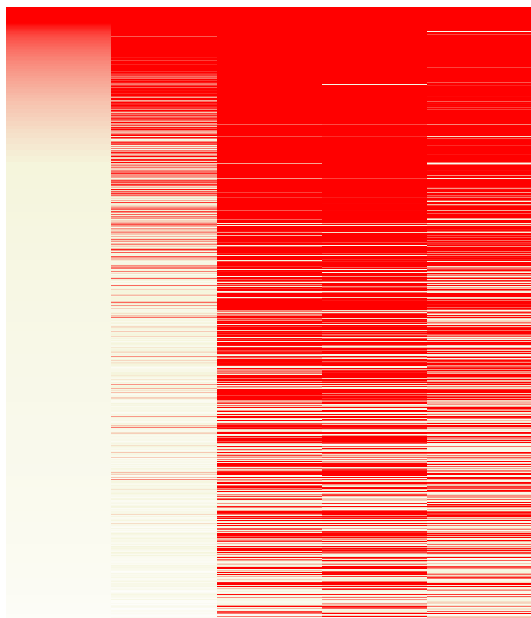

C

P

T1

T2

T3

Supplement: Supplementary file 1 — Supplementary data [file mmc1.zip › figures/ExtDataFig8a_part1.pdf]

$\log_2(\text{Ratio vs C})$

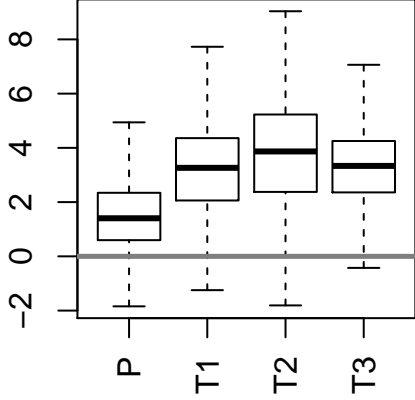

Supplement: Supplementary file 1 — Supplementary data [file mmc1.zip › figures/Fig2f.pdf]

Color Key

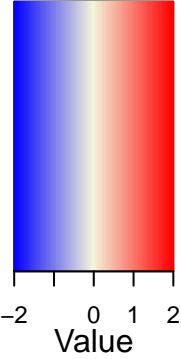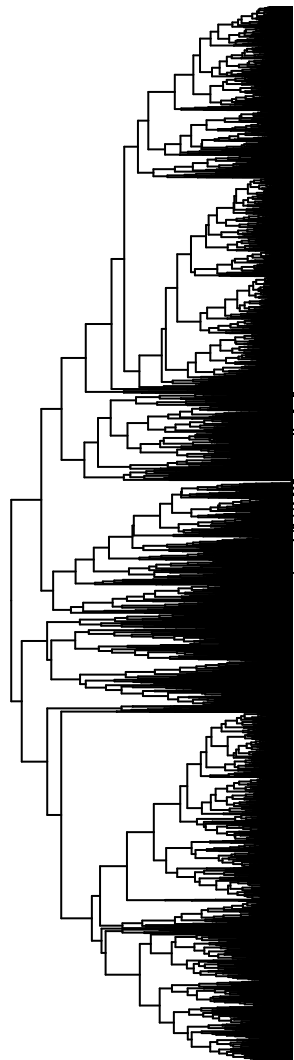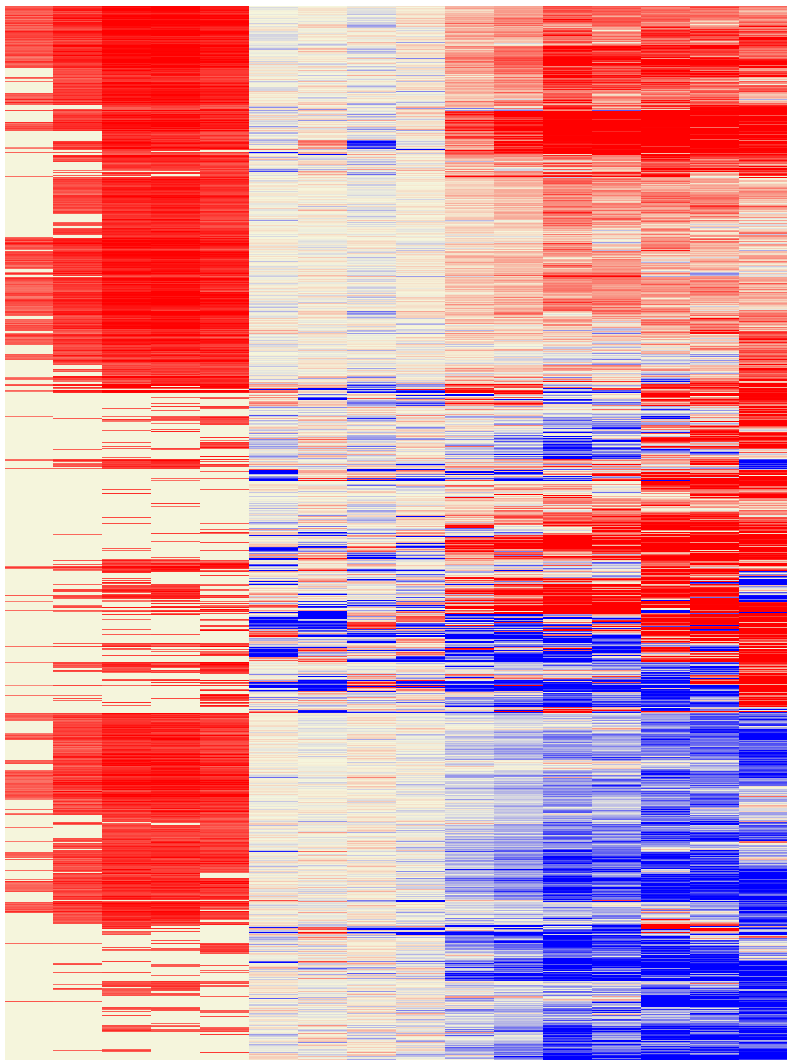

Myc.C1.int  
Myc.P.int  
Myc.T27.int  
Myc.T29.int  
Myc.T31.int  
C\_1  
C\_3  
C\_4  
C\_6  
P\_2  
P\_3  
P\_4  
P\_5  
T\_1  
T\_2  
T\_3

Supplement: Supplementary file 1 — Supplementary data [file mmc1.zip › figures/Fig2a.pdf]

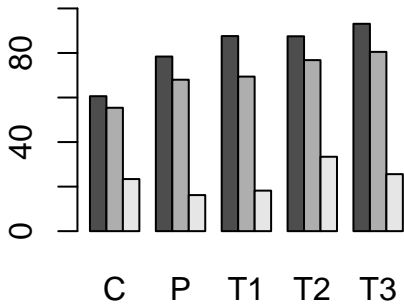

Supplement: Supplementary file 1 — Supplementary data [file mmc1.zip › figures/ExtDataFig2b.pdf]

**Myc.0h 18000**

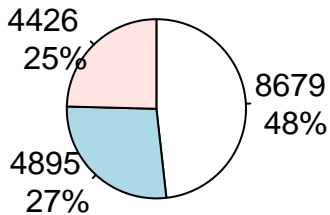

**Myc.4h 27693**

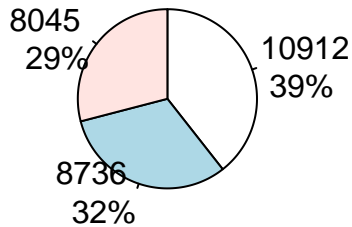

Supplement: Supplementary file 1 — Supplementary data [file mmc1.zip › figures/ExtDataFig7b.pdf]

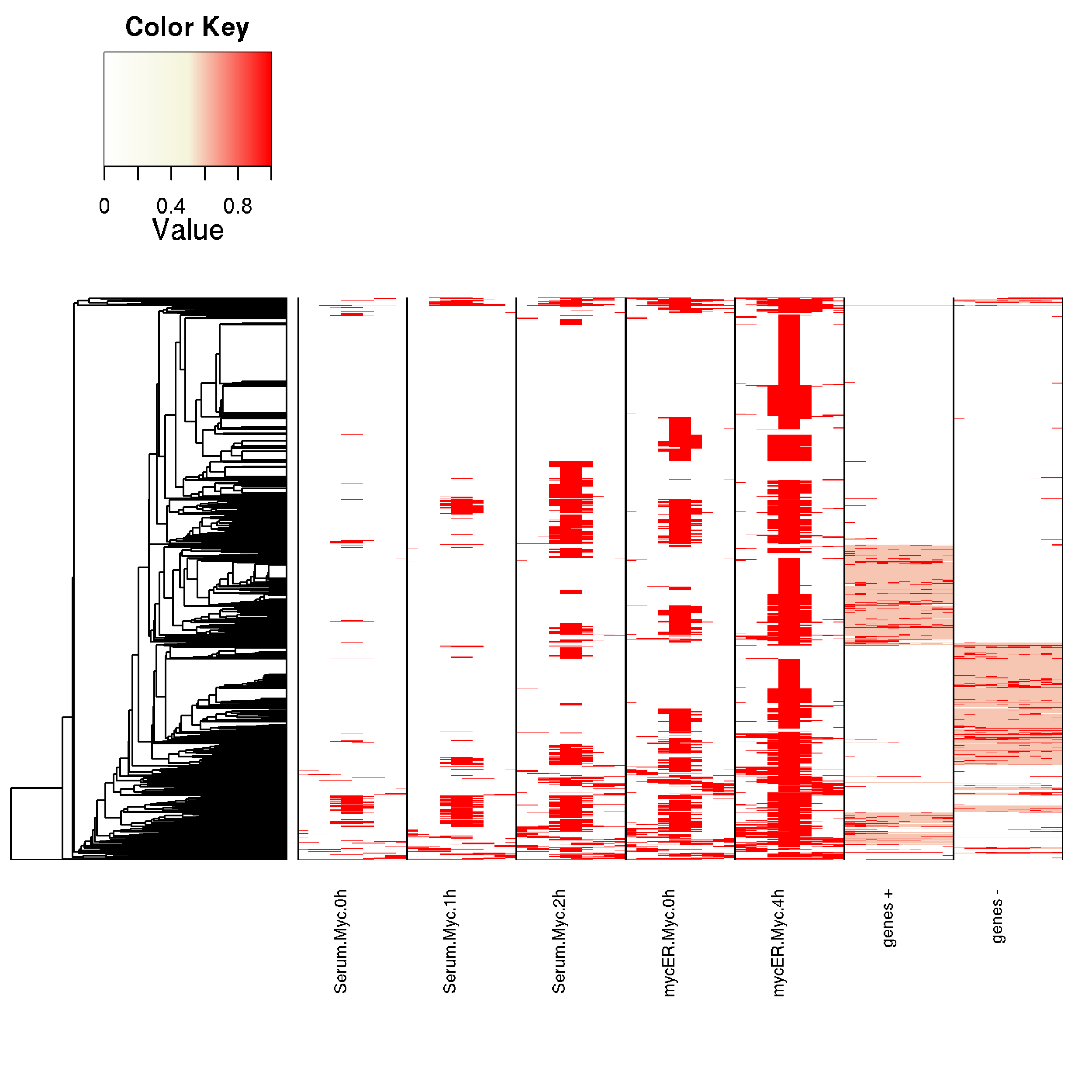

Supplement: Supplementary file 1 — Supplementary data [file mmc1.zip › figures/Fig3h_distal.png]

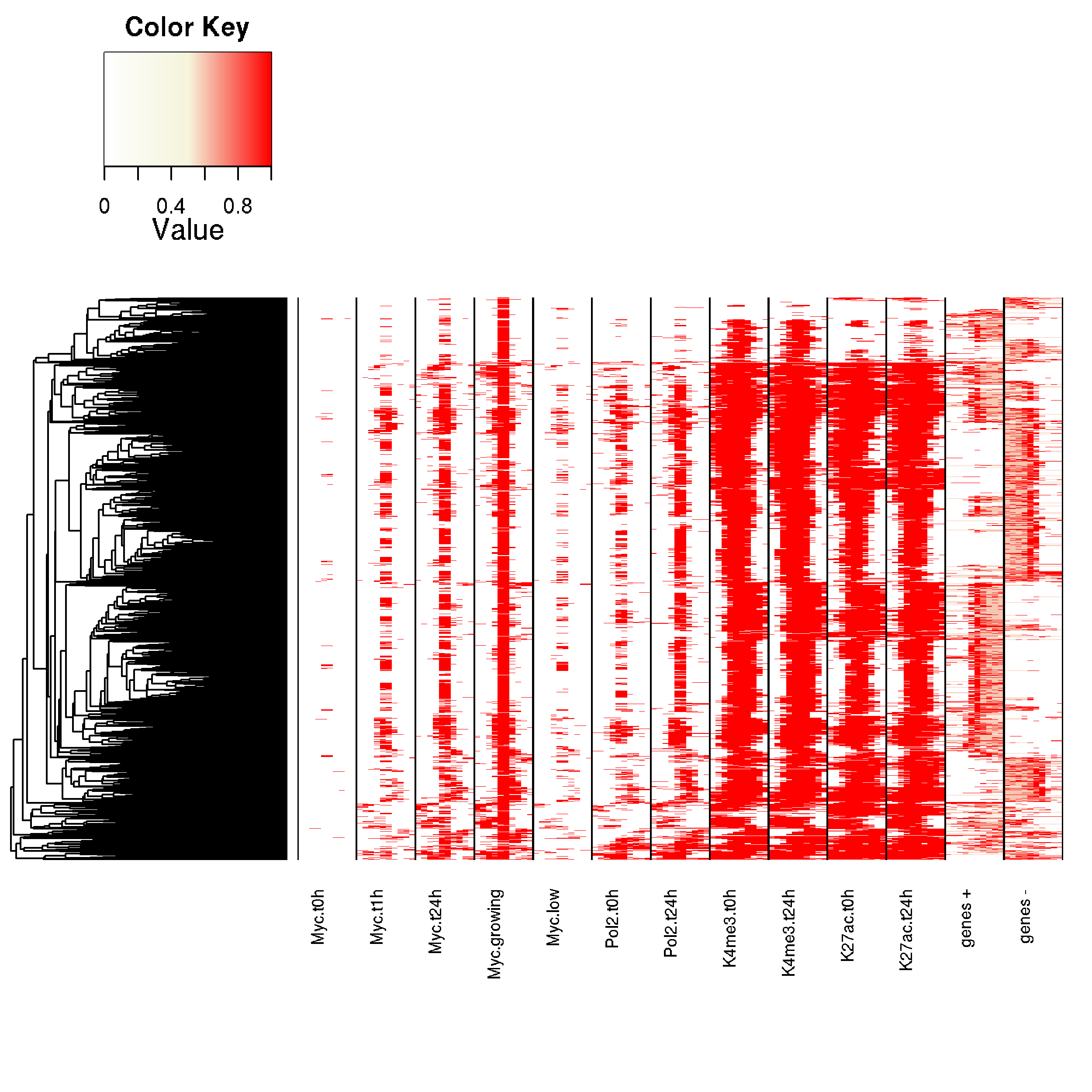

Supplement: Supplementary file 1 — Supplementary data [file mmc1.zip › figures/ExtDataFig6a_part1.png]

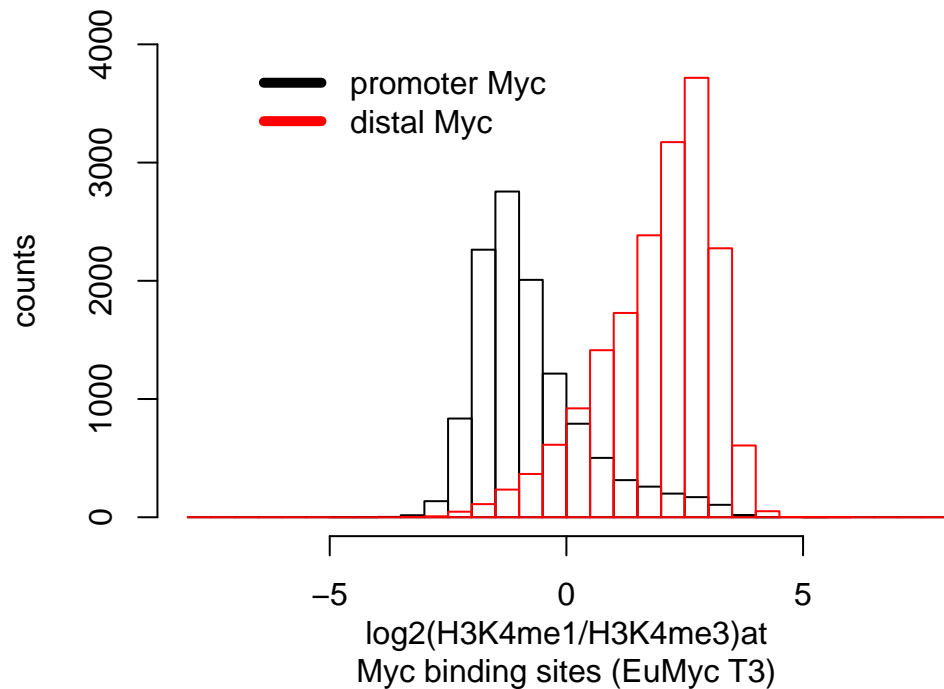

Supplement: Supplementary file 1 — Supplementary data [file mmc1.zip › figures/ExtDataFig2c.pdf]

**Myc.C1 6978**

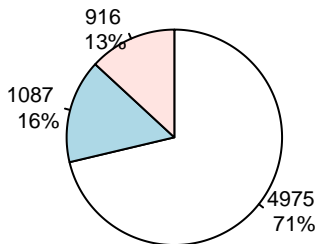

**Myc.T27 29252**

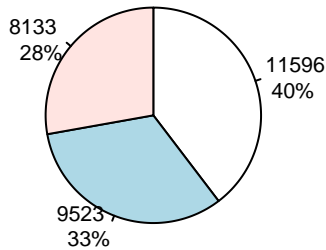

**Myc.T31 34142**

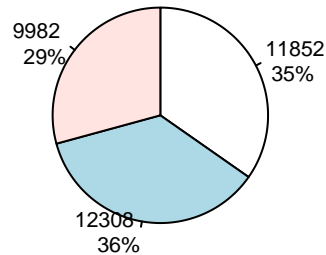

**Myc.P 17340**

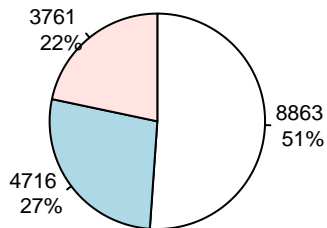

**Myc.T29 27347**

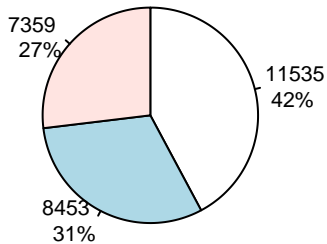

Supplement: Supplementary file 1 — Supplementary data [file mmc1.zip › figures/ExtDataFig1h.pdf]

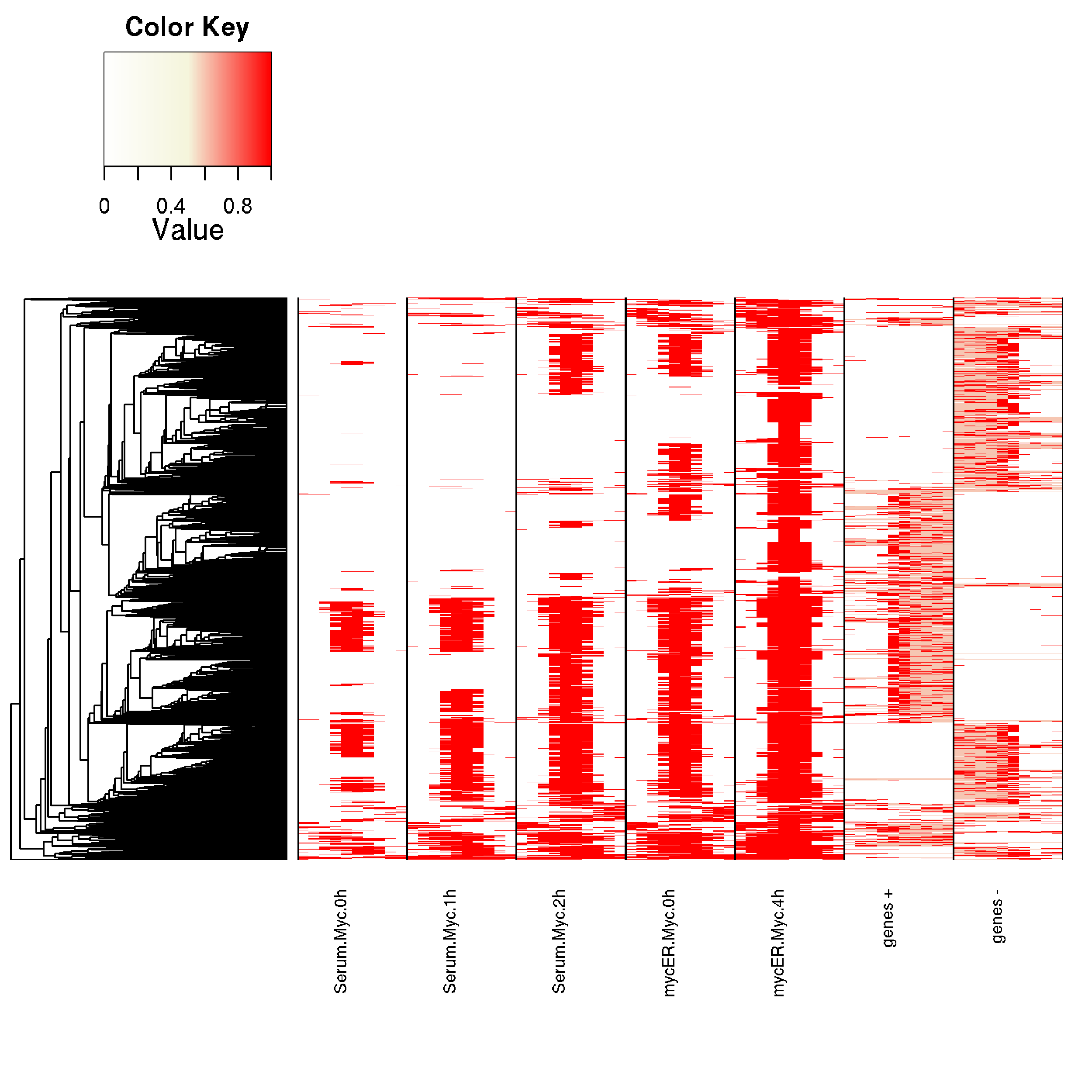

Supplement: Supplementary file 1 — Supplementary data [file mmc1.zip › figures/Fig3h_promoter.png]

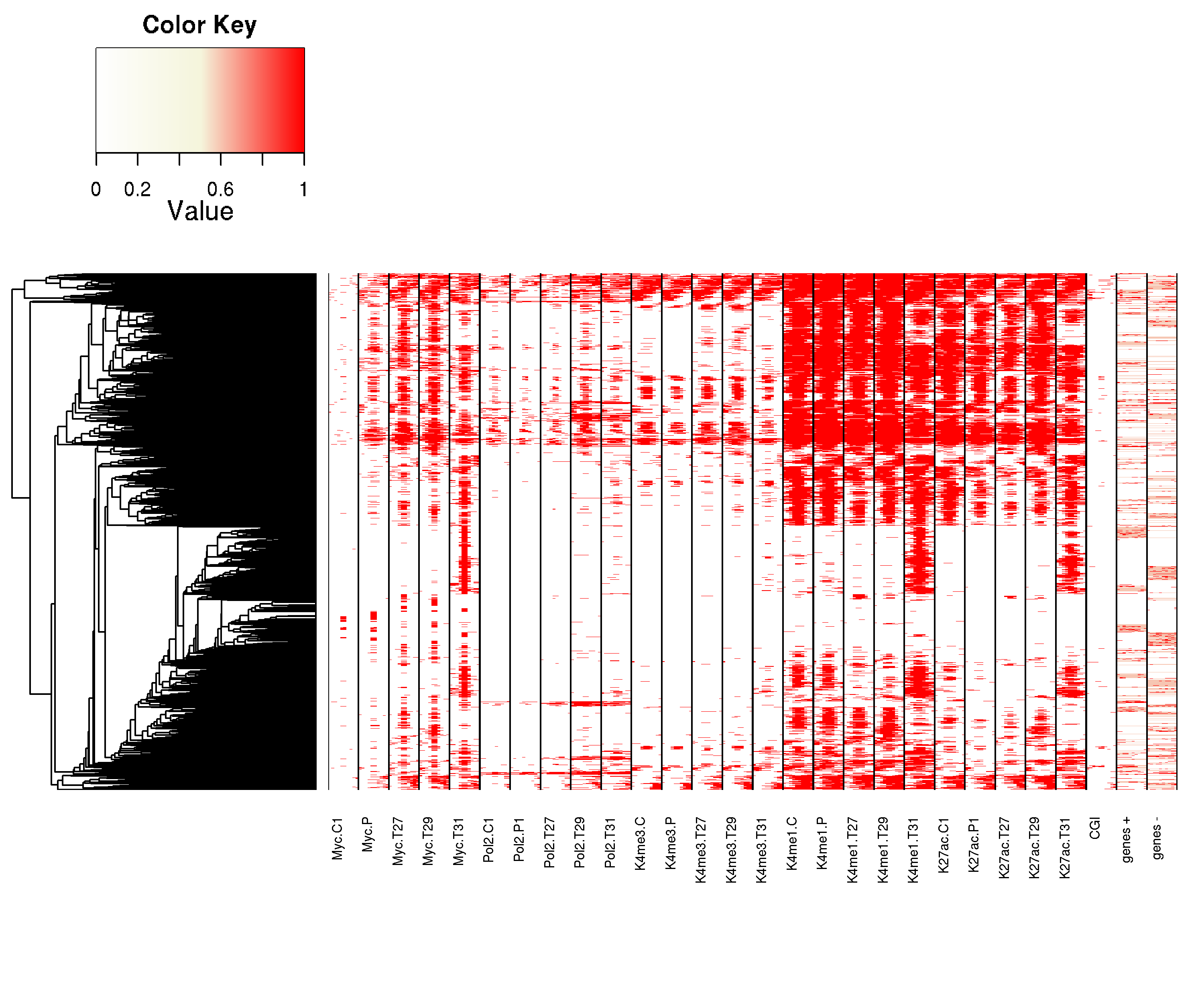

Supplement: Supplementary file 1 — Supplementary data [file mmc1.zip › figures/Fig1b.png]

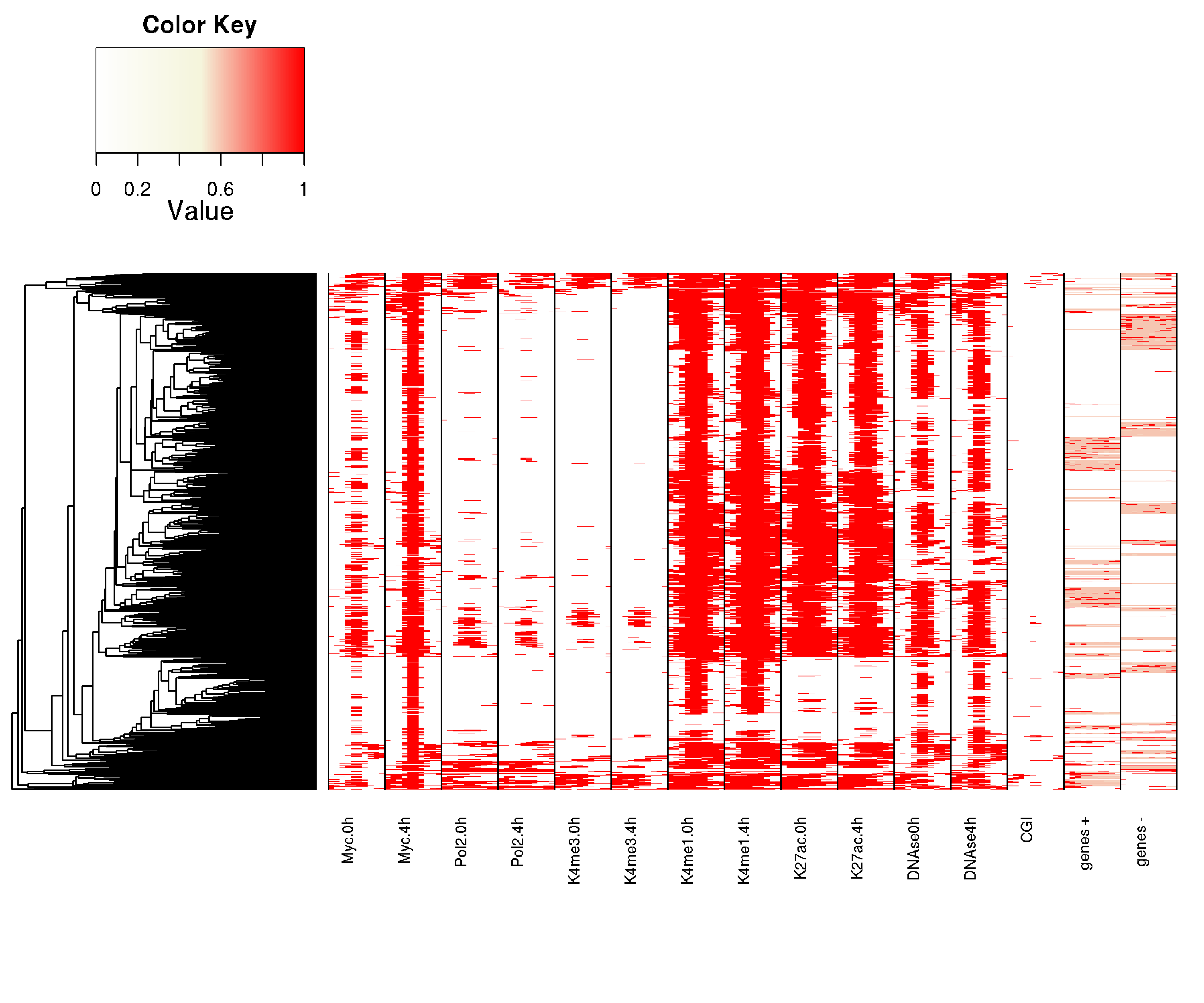

Supplement: Supplementary file 1 — Supplementary data [file mmc1.zip › figures/ExtDataFig7d_part2.png]

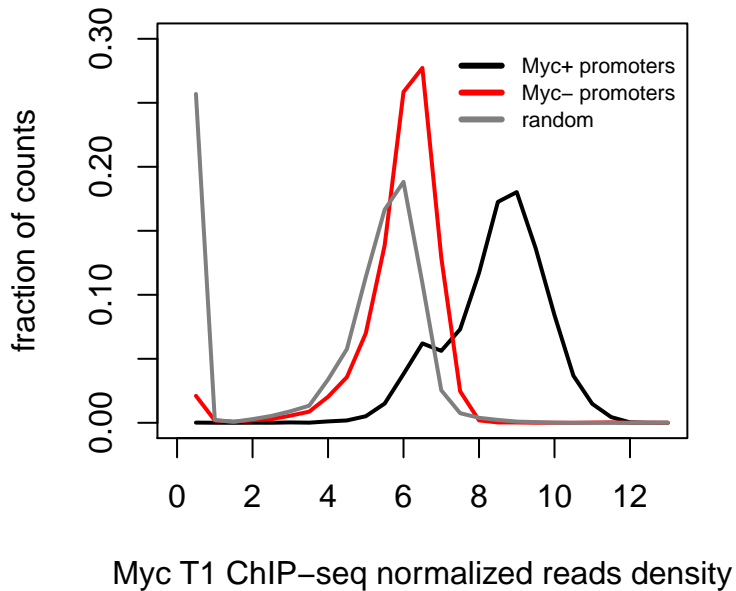

Supplement: Supplementary file 1 — Supplementary data [file mmc1.zip › figures/ExtDataFig1f.pdf]
